# Characterization of *Bacillus velezensis* AK-0 as a biocontrol agent against apple bitter rot caused by *Colletotrichum gloeosporioides*

**DOI:** 10.1038/s41598-020-80231-2

**Published:** 2021-01-12

**Authors:** Young Soo Kim, Younmi Lee, Wonsu Cheon, Jungwook Park, Hyeok-Tae Kwon, Kotnala Balaraju, Jungyeon Kim, Yeo Jun Yoon, Yongho Jeon

**Affiliations:** 1 Department of Plant Medicals, Andong National University, Andong, 36729 Republic of Korea; 2 Central Research Institute, Kyung Nong Co., Ltd., Gyeongju, 38175 Republic of Korea; 3 Department of Microbiology, Pusan National University, Pusan, 46241 Republic of Korea; 4 Agricultural Science & Technology Research Institute, Andong National University, Andong, 36729 Republic of Korea; 5 Research Department, KOREABIO Co., Ltd., Hwaseong, 18514 Republic of Korea

**Keywords:** Applied microbiology, Biotechnology, Microbiology

## Abstract

*Bacillus* genus produces several secondary metabolites with biocontrol ability against various phytopathogens. *Bacillus velezensis* AK-0 (AK-0), an antagonistic strain isolated from Korean ginseng rhizospheric soil, was found to exhibit antagonistic activity against several phytopathogens. To further display the genetic mechanism of the biocontrol traits of AK-0, we report the complete genome sequence of AK-0 and compared it with complete genome sequences of closely related strains. We report the biocontrol activity of AK-0 against apple bitter rot caused by *Colletotrichum gloeosporioides*, which could lead to commercialization of this strain as a microbial biopesticide in Korea. To retain its biocontrol efficacy for a longer period, AK-0 has been formulated with ingredients for commercialization, named AK-0 product formulation (AK-0PF). AK-0PF played a role in the suppression of the mycelial growth of the fungicide-resistant pathogen *C. gloeosporioides* YCHH4 at a greater level than the non-treated control. Moreover, AK-0PF exhibited greater disease suppression of bitter rot in matured under field conditions. Here, we report the complete genome sequence of the AK-0 strain, which has a 3,969,429 bp circular chromosome with 3808 genes and a G+C content of 46.5%. The genome sequence of AK-0 provides a greater understanding of the *Bacillus* species, which displays biocontrol activity via secondary metabolites. The genome has eight potential secondary metabolite biosynthetic clusters, among which, *ituD* and *bacD* genes were expressed at a greater level than other genes. This work provides a better understanding of the strain AK-0, as an effective biocontrol agent (BCA) against phytopathogens, including bitter rot in apple.

## Introduction

The food requirement of the growing human population is very high, and this demand has led to the use of chemical pesticides and fertilizers^1^; however, this strategy has shown adverse effects on environmental and human health and the quality of crop produce^2^. The continuous application of the chemical inputs causes the accumulation of toxic compounds in soils, which are being absorbed by most of the crop plants. The consumption of such crops can lead to health disorders in humans^3^. The application of plant growth-promoting rhizobacteria (PGPR) can help the plant to grow more effectively by developing resistance against various phytopathogens^4^. Biological control by PGPR has emerged as an alternative to reduce the use of conventional agricultural inputs for improving the quantity and quality of crop produce^5^. PGPR localizes in the plant rhizosphere and plays an important role in controlling phytopathogens^6^. In recent years, the yield and quality of many fruit crops have been reduced by attacking various diseases caused by phytopathogens^7–9^; this can be overcome using biological control agents (BCAs).

*Colletotrichum gloeosporioides* causes anthracnose (bitter rot) in several plant species. Several *Colletotrichum* species have been reported to infect a wide range of plant species, including apple^10^. These pathogens can cause two distinct diseases, such as bitter rot and fruit rot in apple^11,12^. Besides, the pathogen causes serious diseases in red pepper, strawberry, and grapevine^13,14^. Lesions of anthracnose appear as small, light brown circles on apple, and later expand, cause killing the host^15^. Anthracnose is distributed primarily in tropical and subtropical areas; however, several prominent species infect temperate crops^16^. Fruit rots are mainly caused by *C. gloeosporioides*, and, to a lesser extent, *C. acutatum*^17^.

*Bacillus velezensis*, an antagonistic bacterium produces several antimicrobial compounds as secondary metabolites^18^ against various phytopathogens. The *Bacillus* sp. produces spores, which can be used for the development of an effective microbial biopesticide formulation in the form of a BCA. Previously, many studies have demonstrated that several secondary metabolites produced by antagonistic bacteria, play key roles in the control of various phytopathogens^19^. Furthermore, *Bacillus* spp., have been found to produce cyclic lipopeptides (CLPs), which play a key role in antimicrobial activity^20^, and can be divided into three main families; surfactin, fengycin, and iturin^21^. Fengycin and iturin are known to exhibit antifungal activities against various phytopathogens, while surfactin exhibits strong antibacterial and antiviral activities^22^. Several species of *B. velezensis* have been reported to produce lipopeptides synthesized by nonribosomal peptide synthetases (NRPSs), which are associated with antifungal activity^23^. Considering these potential benefits, this study aimed to evaluate the potential efficiency of the selected PGPR strain AK-0 as a BCA against apple bitter rot caused by *C. gloeosporioides* under in vitro and field conditions and for plant growth-promotion. Isolation^24^ and identification^7^ of the AK-0 strain were described in our previous studies. To gain an in-depth understanding of the biocontrol ability of AK-0 against apple bitter rot, this study reports the complete genome sequence of AK-0. To further identify the divergent genomic characteristic among other *Bacillus* strains, a comparative genome analysis was performed.

## Results

### Isolation, screening, and identification of antagonistic *Bacillus velezensis* AK-0

Most of the rhizosphere-associated *Bacillus* spp. possess antagonistic activity against several plant pathogens^25,26^. In our study, AK-0 cell suspensions (1 × 10^8^ cfu/mL) exhibited greater antagonistic activity (16 mm) against *C. gloeosporioides* APEC18-004 than other *Bacillus* spp., while there was no antagonism in water-treated control (Fig. S1). The isolate was named *Paenibacillus polymyxa* AK-0 (currently known as *Bacillus velezensis* AK-0). The preliminary characterization of the strain AK-0 was described in our previous study^7^.

### Disease occurrence of bitter rot on apple orchards in northern Gyeongbuk Province in Korea

The symptoms of bitter rot disease caused by *C. gloeosporioides* on apple fruits appeared as a concave-shaped and dark brown on matured apples under field conditions (Fig. S2A). The diseased spots initially appeared as small (2–8 mm in diameter) and later increased in size (10–20 mm in diameter) upon the maturity of fruits. The infections were observed on the fruits where the pathogen emerges after a few weeks and later began as small, slightly sunken, circular, light brown to the dark brown lesions (Fig. S2B). The lesions remained circular and became more sunken as they grew (Fig. S2C). After lesions became 1–3 cm in diameter, acervuli are produced in concentric circles around the point of infection. The microscopic observations revealed that the conidia possessed straight, cylindrical, obtuse, and base truncate (Fig. S2D). The occurrence of disease severity (%) of bitter rot on apple recorded in Gyeongbuk Province during the periods from 2018 and 2019 (Table S4). The highest disease severity (%) was recorded at Jibo-myeon (18.54 ± 2.6) in comparison with other places in the year 2019. Between 2 years, the average disease severity (%) was recorded at a greater level in 2019 than in 2018 due to weather conditions.

### Isolation of pathogenic fungus from the apple orchards and microscopic observation

The fungal pathogen *C. gloeosporioides* was isolated from the symptomatic apple tissues. The average conidial germination percentage ranged from 58 to 70 at 24 h of incubation, and the appressoria formation ranges from 40 to 53% under a light microscope. The colony color, length, width, the shape of conidia, and appressoria of fungal pathogen *C. gloeosporioides* APEC18-004 compared with various *Colletotrichum* spp. Table S5^27^. After the fungal pathogen was inoculated onto the potato dextrose agar (PDA) plate and incubated for 10 days at 25 °C, colonies produced a strong mycelial growth with ideal pigmentation near the center (Fig. S3A). Microscopic observations revealed that conidiomata were acervular, without setae, and measured around 100–200 μm in diameter, with orange conidial masses (Fig. S3B). Conidiophores were subcylindrical and arose directly from the mycelia; tall, slender, and irregularly branched in the terminal portion, and measures 15–45 × 3–4 μm (length × width). They also bore clusters of conidia (Fig. S3C, D). The aerial mycelia of fungus were dense and pale white. The conidia possessed straight, cylindrical, obtuse, and truncate bases in this isolate (Fig. S3E). Furthermore, microscopic observations revealed that the conidia germinated and developed appressoria through a germ tube (Fig. S3F). The average conidial size measured 12.6–18.6 µm × 4.3–5.8 µm (length × width), while the average appressoria size measured 9.2–17.7 µm × 4.5–11.3 µm (length × width) from bitter rot caused by *C. gloeosporioides* APEC18-004 (Table S5).

### Effect of AK-0 bacterial treatment on spore germination of *C. gloeosporioides*

When the conidial spores of *C. gloeosporioides* APEC18-004 were treated with AK-0 bacterial suspensions (10^8^ cfu/mL) under in vitro conditions, the different degrees of damages in conidia germination and germ tube lengths occurred, which were compared to the non-treated control. The conidial spore germination analysis by hemocytometer revealed that there was a greater inhibition percentage of spores after 16 h incubation onwards in AK-0-treated conidia, while the spore germination rate was increased drastically in the non-treated control (Fig. S4A). AK-0-treated conidia of *C. gloeosporioides* APEC18-004 resting on the hard glass surface did not germinate, while water-treated conidia germinated and formed into appressoria through germ tubes at 16 h onwards (Fig. S4B). At 48 h, all the conidia were germinated and appressoria were formed through germ tubes in the water-treated control, suggesting that AK-0 cell suspensions played a role in suppressing the growth of conidial spores of fungal pathogen *C. gloeosporioides* APEC18-004.

### In vitro antagonism and effect of AK-0 cell suspensions treatment on disease suppression of bitter rot caused by *C. gloeosporioides* in matured apples

The in vitro antagonism assay revealed that the AK-0 cell suspensions exhibited antagonistic activity against the fungal pathogen *C. gloeosporioides* APEC18-004 at a greater level in comparison with the non-treated control using a confrontation plate assay (Fig. S5A). To investigate the potential for biocontrol activity (BCA) of AK-0 against anthracnose caused by *C. gloeosporioides* APEC18-004 in vivo, the fresh and healthy apples were treated with bacterial suspensions before fungal pathogen exposure. There was a greater suppression of disease development of *C. gloeosporioides* APEC18-004 in AK-0-treated apple fruits than in the non-treated control (Fig. S5B). The lesion area caused by *C. gloeosporioides* was 1.34 cm in diameter, while in non-treated apples, the lesion area was about fourfold greater than that in AK-0-treated apples. The bacterial suspensions (10^8^ cfu/mL) of AK-0 exhibited an increased level of disease control on the matured apples under in vitro conditions compared to those in the non-treated control, where the diameter of the diseased lesion area reached greater than 7 cm (Fig. S5C). When apples were treated with AK-0 cell suspensions before an artificial inoculation with conidial spores of *C. gloeosporioides* APEC18-004, bitter rot disease was suppressed by 80.7% 5 days after inoculation, compared to that on apples treated with AK-0 cell suspensions after inoculation with fungal spores, where the disease was suppressed by 54.9% (Fig. 5S,D). Therefore, the disease incidence was controlled at a greater level in pre-treatment of AK-0 cell suspensions than post-treatment.

### Selection of fungicide-resistant strain *C. gloeosporioides* APEC18-004 and inhibition of conidial germination by AK-0 cell suspensions and its culture filtrate

Out of 19 fungal pathogenic isolates of *C. gloeosporioides* tested for resistance to chemical fungicides (pyraclostrobin and tebuconazole) under in vitro conditions, two fungal isolates namely, YCHH4 and AWKM13, exhibited resistance to pyraclostrobin and tebuconazole, respectively, on PDA plates (Fig. S6A,B). Both fungal strains (YCHH4 and AWKM13) exhibited mycelial growth 10-days after incubation at a greater level in PDA plates amended with commercial fungicides, therefore, the two strains most resistant to each of the antifungals was selected, while the remaining fungal isolates of *C. gloeosporioides* exhibited moderate resistance to chemical fungicides compared to that in the non-treated control.

The effects of AK-0 bacterial suspensions and its culture filtrate (CF) interaction on conidia germination and hyphal morphology of fungicide-resistant isolate YCHH4 were visualized by light microscopy. When conidial spores of YCHH4 were treated with AK-0 bacterial suspensions at various concentrations (10^6^, or 10^7^, or 10^8^ cfu/mL) and CF as well under in vitro conditions, different degrees of damage in conidia germination occurred, which were compared to that in the non-treated control (Fig. S7). The conidial spore germination by hemocytometer analysis revealed that the spore germination was arrested by treatment with AK-0 bacterial suspensions at 10^8^ cfu/mL after 24 h of the incubation period, while the other two concentrations (10^6^ and 10^7^ cfu/mL) did not arrest the spore germination; furthermore, the spore germination rate was increased drastically in the non-treated control. In the CF-treated conidia, the spore germination was arrested as it occurred by treatment with AK-0 cell suspensions (10^8^ cfu/mL) after 24 h of incubation. At 24 h, all conidia were germinated and formed appressoria through germ tubes in the water-treated control.

### Plant growth-promoting effect

The isolate, AK-0, was further assayed for indole-3-acetic acid (IAA) detection and the plant growth-promoting (PGP) activity. The strain AK-0 was found to be effective for IAA detection in comparison to the non-treated control (Fig. S8A). Similarly, the PGP ability of AK-0 on red-pepper seedlings under greenhouse conditions was found to be greater in terms of plant height compared to that in the non-treated control (Fig. S8B). The ability of AK-0 for in vitro IAA detection was effective for plant growth-promotion.

### In planta and in vitro suppression of apple bitter rot and conidial germination of *C. gloeosporioides* by AK-0 product formulation (AK-0PF)

**Figure 1:**
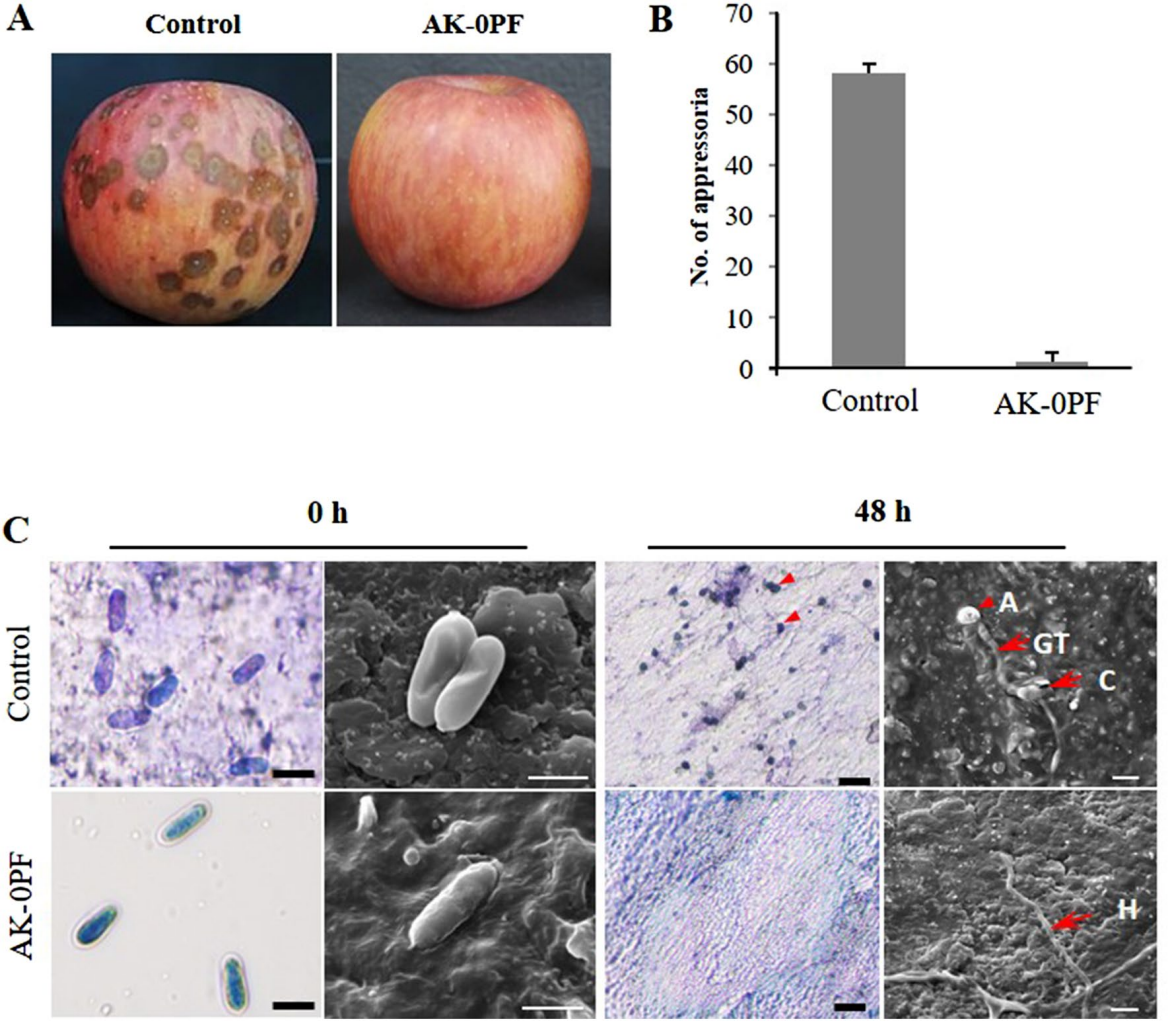
The ability of AK-0 product formulation (AK-0PF) to control anthracnose disease caused by *Colletotrichum gloeosporioides* and microscopic observation. (**A**) Disease suppression of anthracnose caused by *C. gloeosporioides* APEC18-004 on matured apples treated with AK-0PF. (**B**) Conidial germination was suppressed by AK-0PF. (**C**) Microscopic observations and scanning electron microscope (SEM) analysis of conidial germination and appressorium formation of *C. gloeosporioides* after AK-0 formulation treatment during 48 h of incubation at 25 ℃ compared to that in the non-treated control. *A* appressorium, *GT* germ tube, *C* conidium, *H* hyphae. Bar = 10 µm (microscopy), bar = 20 µm (SEM). The experiment was repeated at least once.

The AK-0PF exhibited a greater biocontrol efficacy of bitter rot on the matured apples than did the non-treated control (Fig. 1A). Similarly, the conidial germination and appressoria formation were suppressed by AK-0PF after 48 h of incubation at 25 ℃ compared to those in the non-treated control, where there was an increased appressoria formation from the germinated conidia of *C. gloeosporioides* (Fig. 1B). In addition to the biocontrol assay, AK-0PF is known to suppress the development of fungal growth. Moreover, the microscopic analysis revealed that there was no conidia germination nor appressoria formation when spore suspensions were treated with AK-0PF, while these did occur in the non-treated control at 48 h (Fig. 1C), suggesting that AK-0PF can be efficiently used under field conditions to control apple bitter rot.

### Effect of AK-0PF and chemical fungicide applications on disease incidence, and chlorophyll content under field conditions

**Figure 2:**
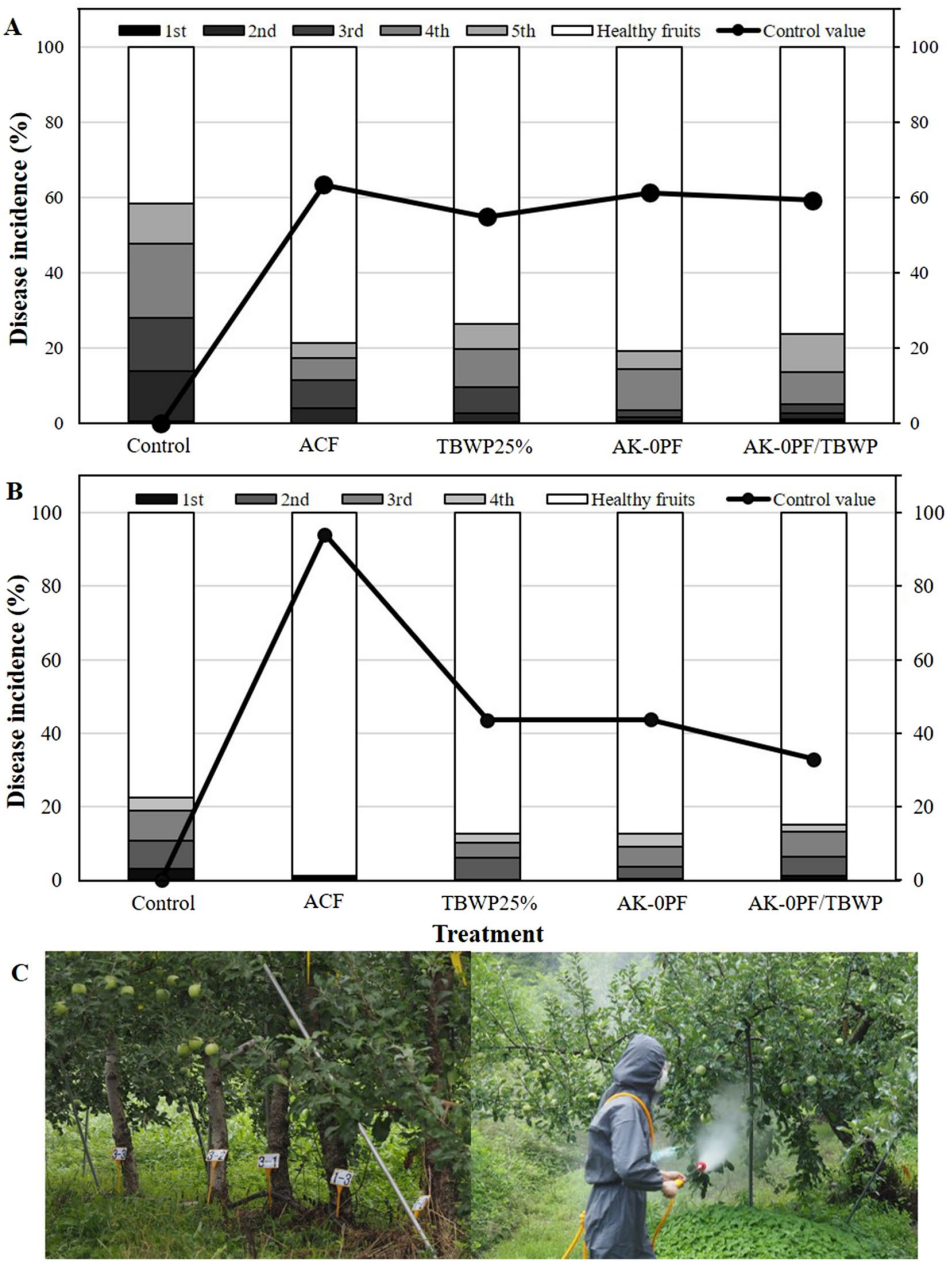
Effect of AK-0 product formulation and chemical fungicide application against bitter rot caused by *Colletotrichum gloeosporioides* on apple orchards (under field conditions) in Yecheon (**A**) and Mungyeong (**B**) in Gyeongbuk Province, Korea. (**C**) Representative photographs of the apple orchard showing the application of AK-0PF (100-fold) during the period from July 2019 to October 2019 to control apple bitter rot. A total of nine times applications was performed by the foliar spray method using an electric power sprayer. Each treatment contained three replicates (plants) in the experiment. Results were compared using the least significant difference (LSD) test ‘R’, with *P* < 0.01. The diseased fruits were harvested five and four times, in Yecheon and Mungyeong, respectively, to record the incidence (%). Control values (disease control %) are expressed along with the data. Control: plant treated with water, ACF: plants treated with agricultural chemical fungicides (Table S1), TBWP: plants treated with tebuconazole 25% WP (wettable powder), AK-0PF: AK-0 product formulation, AK-0PF/TBWP: plants treated with AK-0PF or TBWP as an alternative spray every week. The total number of diseased fruits harvested corresponding to 100% were 492, 649, 788, 1353, and 1087 for control, ACF, TBWP25%, AK-0PF, and AK-0PF/TBWP25, respectively in Yecheon, while the diseased fruits were 323, 450, 510, 385, and 383 for control, ACF, TBWP25%, AK-0PF, and AK-0PF/TBWP25, respectively in Mungyeong.

Based on the in vitro results on disease suppression by AK-0PF, we tested the ability of AK-0PF to suppress apple bitter rot caused by *C. gloeosporioides* under field conditions at two different locations (Yecheon and Mungyeong) in Korea. The disease incidence (%) was drastically reduced as it occurred through agriculture chemical fungicides (ACF), where all the treatments were included either fungicides or insecticides (Fig. 2, Table S1). In Yecheon, because the orchards are in a mountain region, there was an accumulation of smog in the treatment and hence the disease incidence (%) was found to be greater (Fig. 2A) than the disease incidence (%) from apple orchards in Mungyeong (Fig. 2B,C), where the orchard field is exposed to sunshine in a low altitude area. The disease incidence (%) was reduced at a greater level in AK-0PF treatment when compared with other treatments in Yecheon, while the disease incidence in AK-0PF treatment was found to be similar with treatments of TBWP25% and AK-0PF/TBWP in Mungyeong; however, all treatments exhibited disease suppression effect in comparison to the non-treated control. On the other hand, the control value in agricultural chemical fungicides (ACF) treatment was found to be higher with no diseased fruits when compared to all other treatments. These results suggest that AK-0PF is a potential candidate for the biological control of bitter rot disease in apple orchards in Korea. The AK-0PF treatment further contributed to increasing the chlorophyll content at a minor level when compared to other treatments under field conditions in both Yecheon (Fig. S9A) and Mungyeong (Fig. S9B).

### General genomic features of *B. velezensis* AK-0 and comparative analysis with other *Bacillus* strains

**Figure 3:**
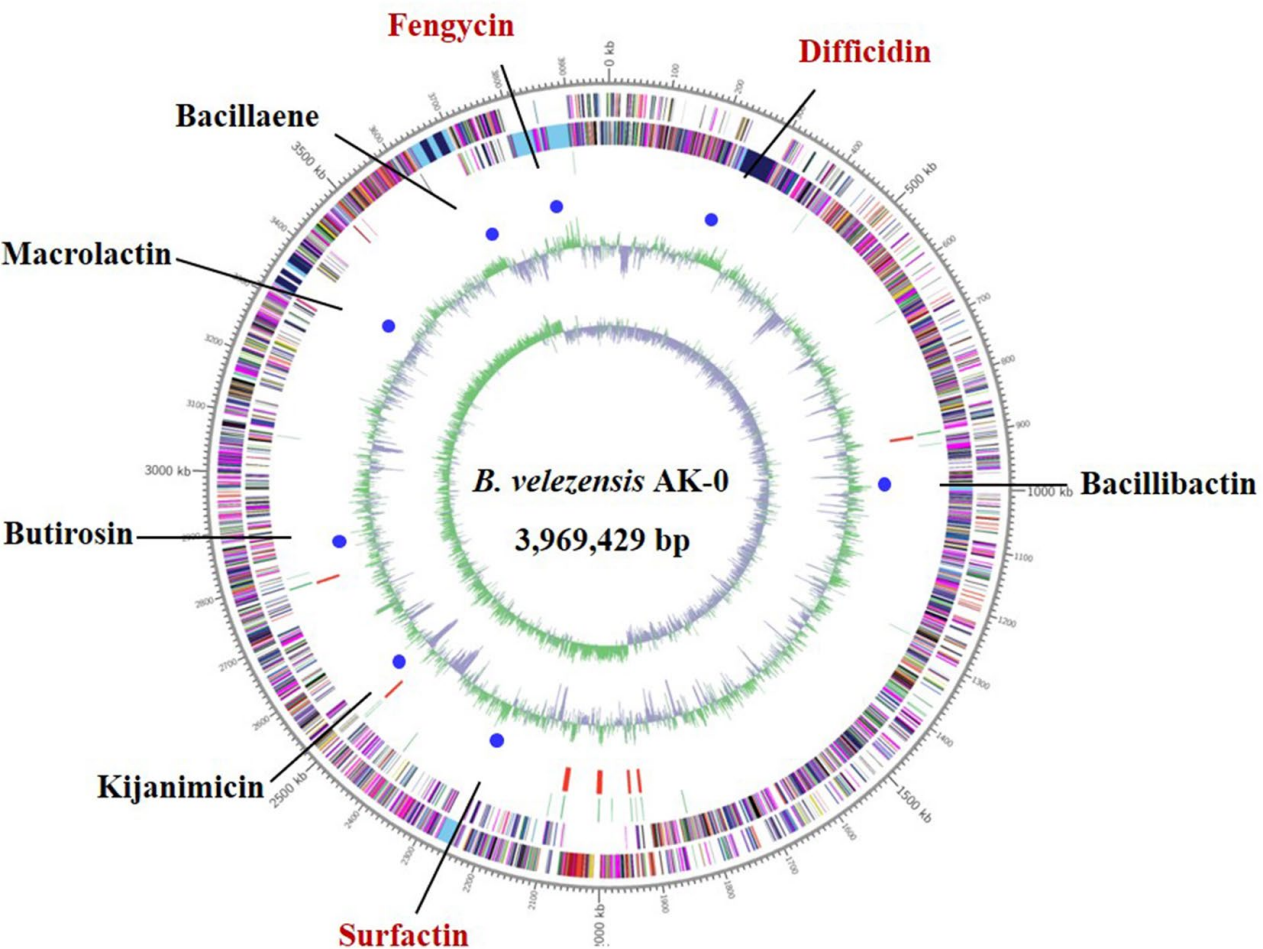
Whole-genome map of *Bacillus velezensis* AK-0. Marked characteristics are shown from the outside to the center; coding sequence (CDS) on the forward strand, CDS on the reverse strand, tRNA, rRNA, guanine-cytosine (GC)-content, and GC skew. Secondary metabolites with biocontrol activity were also displayed on this ring using blue arcs (surfactin, fengycin, bacillibactin, macrolactin, bacillaene, difficidin, kijanimicin and butirosin).

**Figure 4:**
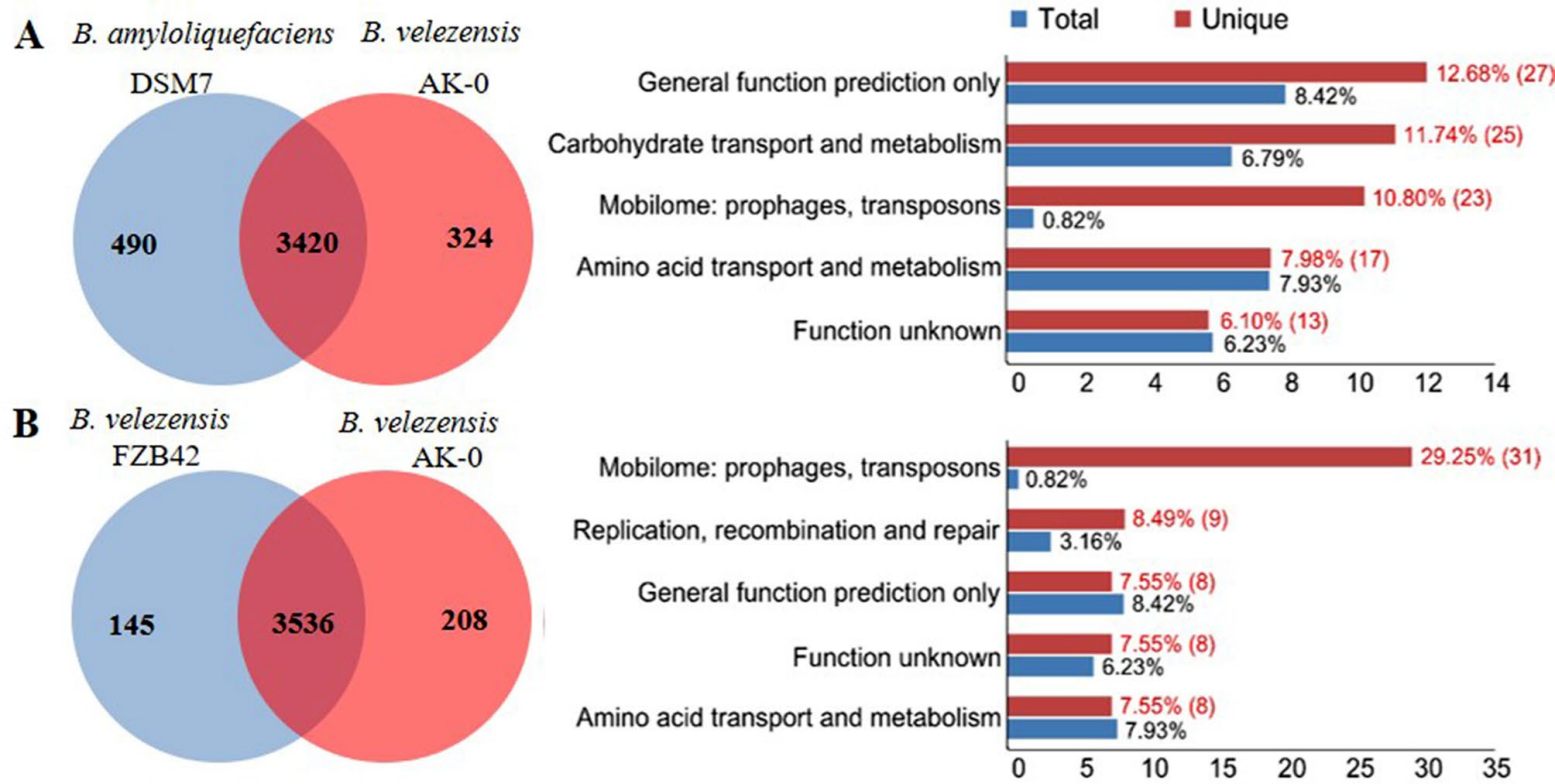
Venn diagram showing the comparison of common sharing unique protein-encoding genes between *Bacillus velezensis* AK-0 and *B. amyloliquefaciens* DSM7 (**A**), and *B. velezensis* FZB42 and *B. velezensis* AK-0 (**B**). A summary of unique SNPs from the total genes of the *B. velezensis* strain is shown here. The numbers 3420 and 3536 indicate the number of common high-expression gene families between the two strains DSM7 and AK-0 and between the other two strains FZB42 and AK-0, respectively.

The genome of the *B. velezensis* AK-0 (AK-0) strain consisted of a circular chromosome of 3,969,429 bp, with 3808 predicted protein-coding sequences (CDSs) in 3909 genes, 86 tRNA genes, 27 rRNAs, and an average G+C content of 46.5% based on NCBI Prokaryotic Genomes Automatic Annotation Pipeline (PGAAP) analysis (Fig. 3, Table S6). The full genome sequence has been deposited in NCBI under the GenBank accession number CP047119. A comparative analysis between the genome sequences of the strain AK-0 and twenty other *Bacillus* strains showed a similar genome (Table S6). Most of the genes in AK-0 were associated with secondary metabolite biosynthesis, transport, metabolism of amino acids, carbohydrates, and catabolism. The genome of the AK-0 strain was compared with four closest known evolutionary relatives: *B. velezensis* UCMB5036 (GCA_000341875.1), *B. velezensis* AS43.3 (GCA_000319475.1), *B. velezensis* FZB42 (GCA_000015785.1), and *B. amyloliquefaciens* DSM7^T^ (GCA_000196735.1). Venn diagrams (Fig. 4) show the comparison of common CDSs between *B. velezensis* AK-0 and *B. amyloliquefaciens* DSM7, and between *B. velezensis* FZB42 and *B. velezensis* AK-0. A summary of unique protein-encoding genes from the total genes of *B. velezensis* strain is shown here. The number of common high-expression gene families shared between DSM7 and AK-0 and between FZB42 and AK-0 were identified as 3420 (Fig. 4A) and 3536 (Fig. 4B), respectively, while 324 and 208 genes were determined to be unique for AK-0 when shared with DSM7 and FZB42, respectively. A phylogram based on computing of the *Bacillus* core genomes suggested a close relationship with other *Bacillus* strains (Fig. S10). Furthermore, to expand our understanding of the metabolic pathways related to antagonism in AK-0, the enrichment analysis module in MetaboAnalyst was used, which verified that metabolism was significantly associated with AK-0, which shows 11 metabolites that were associated with antagonism (Fig. S11A). The metabolic network of the differential metabolites and altered metabolic pathways in the KEGG general metabolic pathway map is shown in Fig. S11B.

### Function and classification of genome annotation

**Figure 5:**
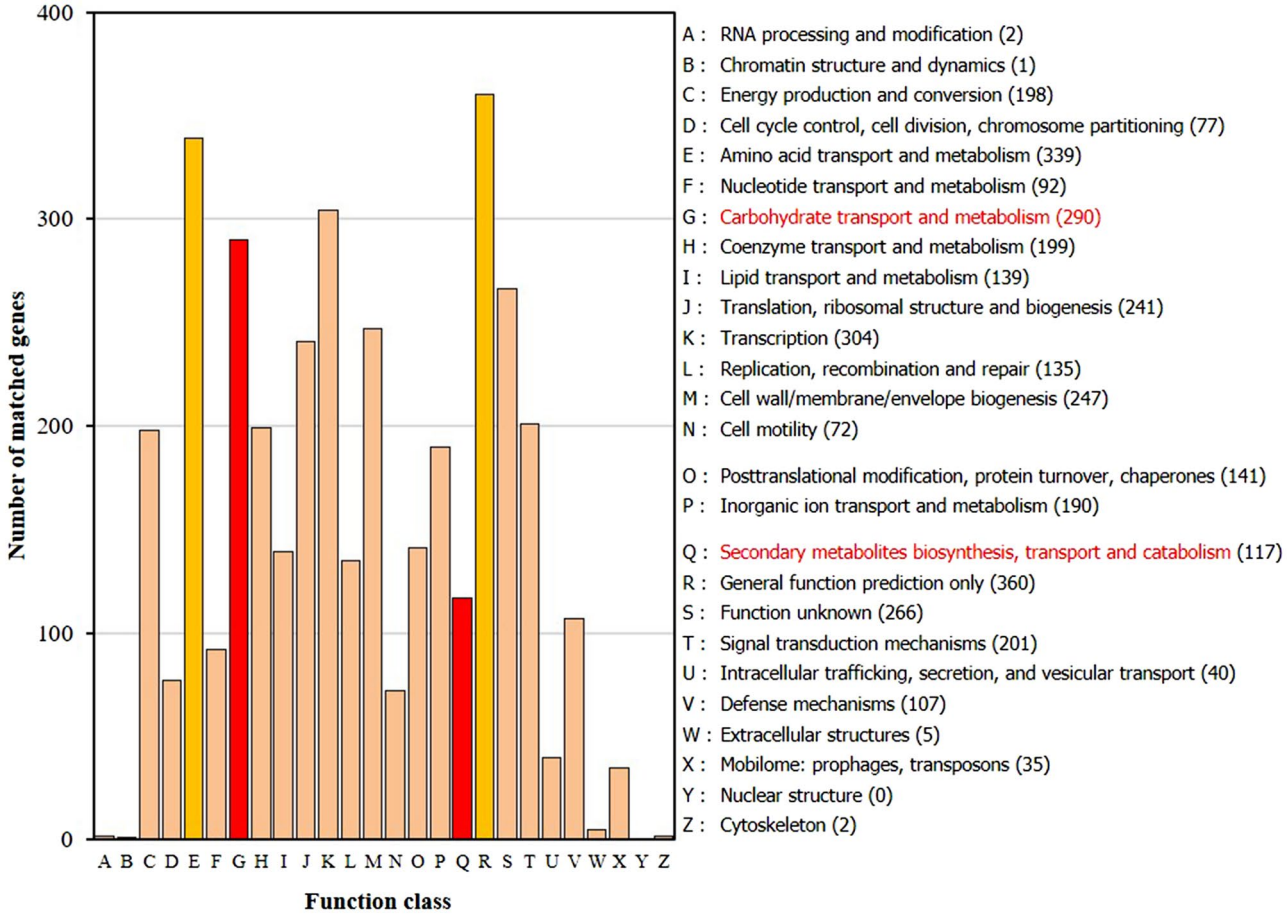
The COG function annotation of *Bacillus velezensis* AK-0. Distribution of genes in different COG function categories.

All the predicted protein sequences of AK-0 were compared to those in the COG database to identify the homologous amino acid sequences in the database. Each protein was assigned with a COG number when functionally annotated and represented a class of protein, then the proteins were subjected to functional clustering analysis according to the COG function (Fig. 5). According to the results of the COG annotation, 3808 proteins in AK-0 were classified into 18 COG families (Table S7). The largest group of genes was involved in general function prediction (360 genes) and amino acid transport and metabolism (339 genes). A total of 117 proteins were involved in secondary metabolite biosynthesis, some of which were involved in amino acid transport and metabolism (E), transcription (K), and carbohydrate transport and metabolism (G). Therefore, AK-0 synthesized high amounts of secondary metabolites, especially antibiotic substances.

### Analysis of biosynthetic gene clusters (BGCs) of CLPs in *B. velezensis* AK-0

**Figure 6:**
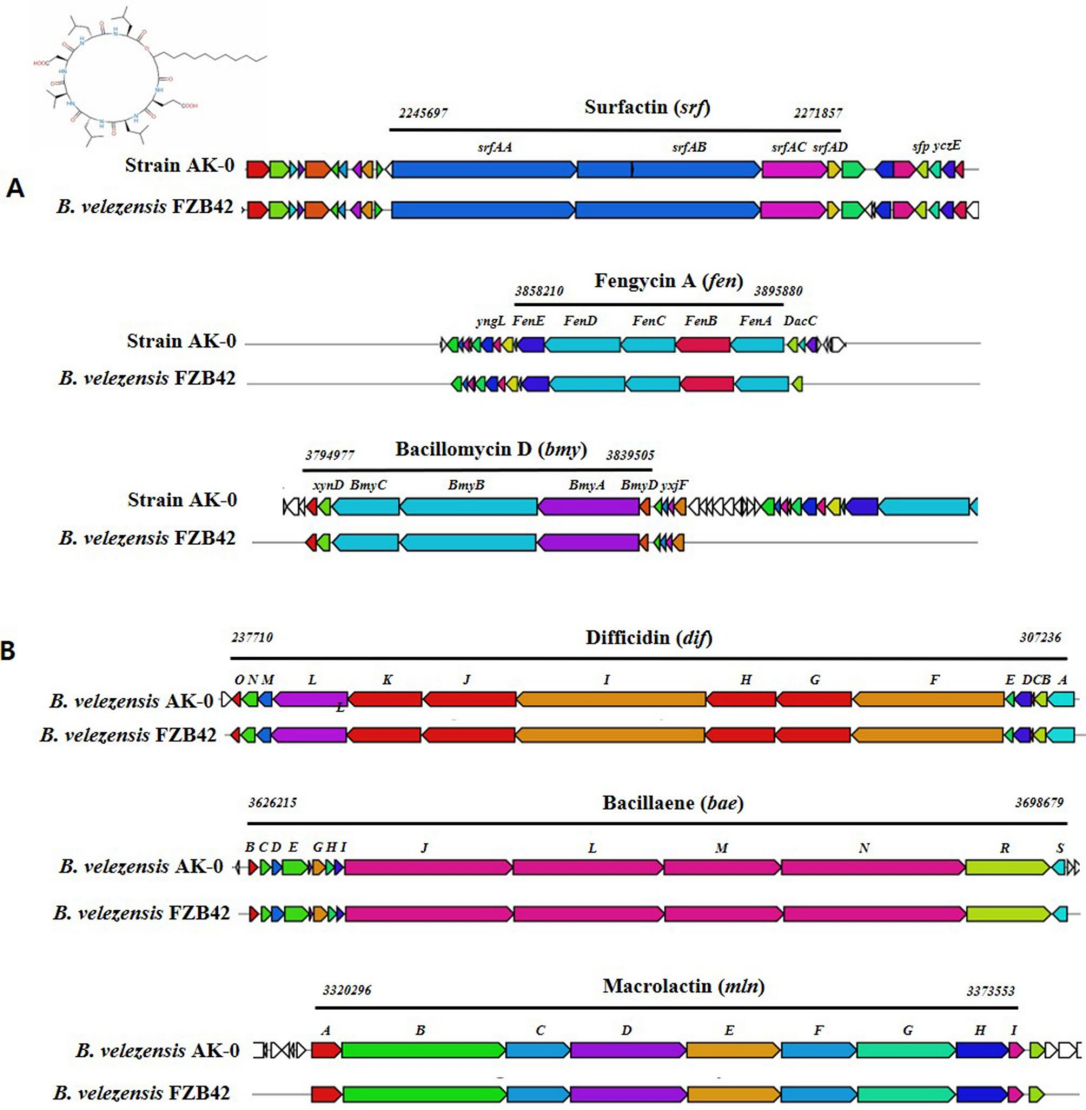
Secondary metabolite gene clusters with antimicrobial metabolites in *Bacillus velezensis* AK-0. (**A**) Secondary metabolite gene clusters with antifungal metabolites (surfactin A, fengycin A, and bacillomycin). (**B**) Secondary metabolite gene clusters with antibacterial metabolites (difficidin, bacillaene, and macrolactin). Arrows indicate gene clusters. Genes highlighted by various colored arrows represent the core biosynthetic genes.

According to the antibiotics and secondary metabolite analysis shell (antiSMASH4.0) analysis of the AK-0 genome, 30 gene clusters were involved in the secondary metabolism of the strain, and 3 gene clusters were involved in the synthesis of CLPs via NRPSs: surfactin (*srf*), fengycin (*fen*), bacillomycin (*bmy*), with antifungal activities (Fig. 6A). Further, the gene clusters are likely to be involved in the secondary metabolites of difficidin (*dif*), bacillaene (*bae*), and macrolactin (*mln*) possessing antibacterial activities (Fig. 6B).

In a further analysis of biosynthesis of the surfactin gene cluster, it consisted of a seventh module with the classical C-A-T tridomain architecture, a termination Te domain. and an ACPS domain. Three gene clusters with polyketide synthase (PKS) genes in the AK-0 genome are involved in the biosynthesis of bacillaene (bae) and difficidin (dfn). Two other clusters for the antibiotics plantathiazolicin and butirosin synthesized from other *Bacillus* strains were found in the AK-0 genome (Table S8). Furthermore, an NRPS cluster of eight genes that may encode a novel antibiotic was found in the chromosome of AK-0. As a result, AK-0 shows great capability for antibiosis. The sequences corresponding with the production of nonribosomal peptides, such as surfactin, bacillibactin, and fengycin, matched to 91%, 100%, and 100% of the identified gene clusters, respectively (Table S8), suggesting that AK-0 could produce a new kind of surfactin.

### Detection of genes for secondary metabolites from *B. velezensis* AK-0 using qPCR

**Figure 7:**
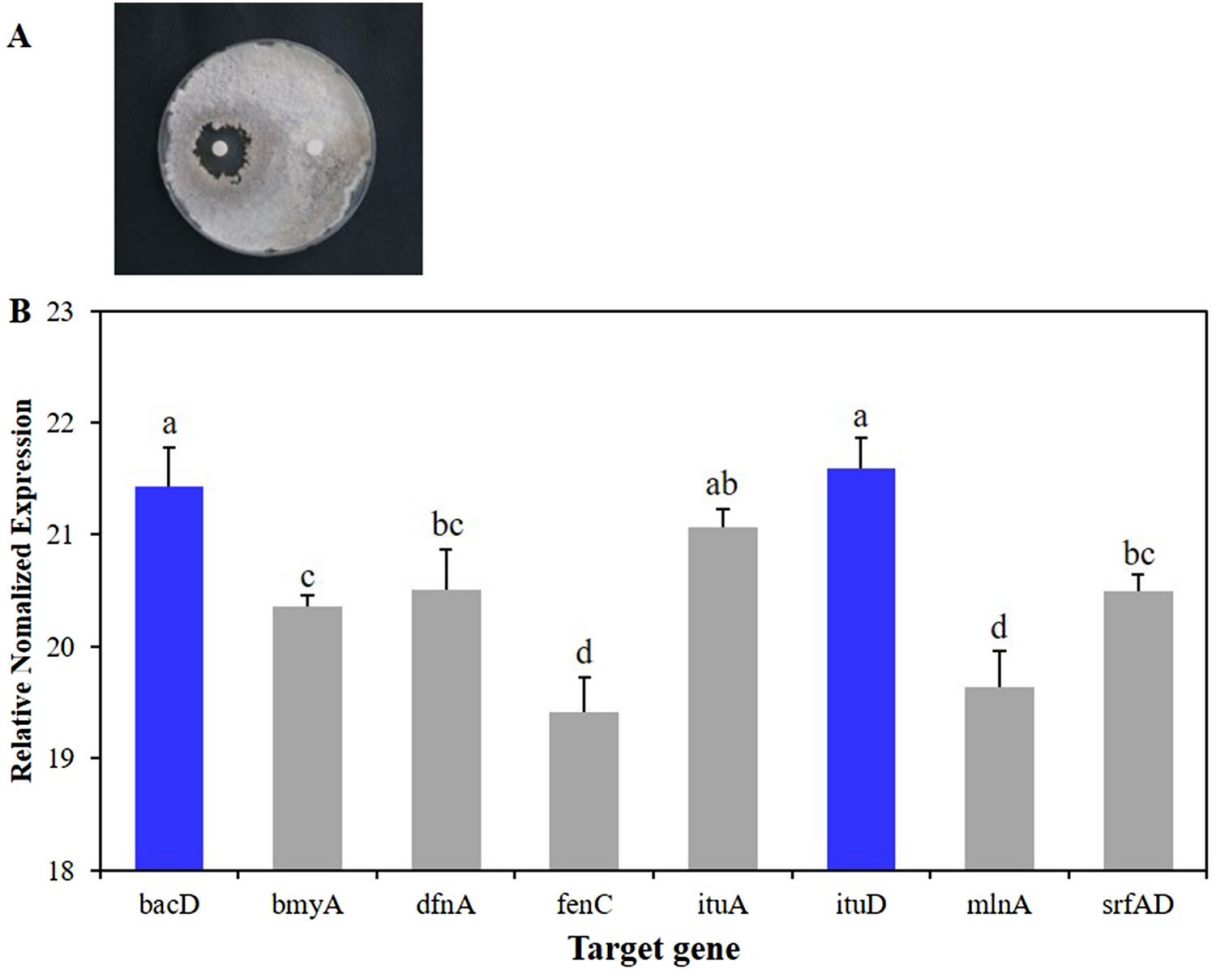
Gene expression analysis of secondary metabolites from the *B. velezensis* AK-0 genome using qPCR. (**A**) Secondary metabolites produced by AK-0 when interacted with a pathogen by producing an inhibition zone (**B**) Among the eight genes, the *ituD*, and *bacD* genes were expressed at a greater level than other genes from the *B. velezensis* AK-0 genome.

The AK-0 cell suspensions exhibited a greater antagonistic activity, which indicates the ability of AK-0 to produce secondary metabolites when interacting with a pathogen (Fig. 7A). AK-0 possessed eight secondary metabolite clusters within the genome, among which the *bacD* and *ituD* genes were expressed at a greater level in comparison with the expressions of other secondary metabolites (Fig. 7B). The genome contained five NRPS clusters, which were conserved in all *B. velezensis* members.

## Discussion

Most chemical controls are ineffective against fungal diseases, owing to the development of resistance to chemical fungicides^26,28^. *Bacillus* species that produce antibiotic compounds as secondary metabolites could be a solution in plant disease management. Here, we isolated the Korean ginseng rhizosphere-associated *B. velezensis* strain AK-0, which exhibited potent antagonistic activity against apple bitter rot caused by *C. gloeosporioides* both under in vitro and field conditions.

Recently, a few other *Bacillus* species have been found to have antifungal activity against *C. gloeosporioides*^19,29^ and *Botryosphaeria dothidea*^30^, which cause anthracnose and apple ring rot diseases, respectively. Anthracnose (bitter rot) caused by *Colletotrichum* species can lead to considerable damage in various crops, including apple^31^, suggesting that infection was due to cross-contamination. *Colletotrichum* spp. are frequently associated with several apple diseases worldwide^32^, whose morphological characteristics are similar to those of *Colletotrichum nymphaeae*^33^. However, the control of apple bitter rot by rhizobacteria is little studied. To the best of our knowledge, this study is the first report on the use of the AK-0 strain as a potential BCA of apple bitter rot disease, and the first analysis of the whole-genome sequencing of AK-0. The results of our study improve our understanding of the biocontrol mechanism of the strain AK-0 and provide a possible alternative BCA to control apple bitter rot. Furthermore, the AK-0 culture filtrate (CF) showed antifungal effects against several fungal phytopathogens, including *C. gloeosporioides*, in our previous study^7^.

On the other hand, treatment with AK-0 CF was equally effective among cell suspensions to inhibit the conidial germination of *C. gloeosporioides* (Fig. S7). Similarly, Khan et al*.*^34^, recently reported that the CFs from *Bacillus* spp. exhibited significant antifungal activity against *Fusarium* spp., while other studies have reported that the CFs of *Gliocladium virens*^35^ and *Trichoderma harzianum*^36^ inhibited the growth and spore germination of *Botrytis cinerea* through chitinolytic glucanolytic, cellulolytic and xylanolytic enzymes.

Previously, a study by Han et al*.*^19^ stated that the conidial germination of *C. gloeosporioides* was inhibited by four *B. atrophaeus strains*, of which the *B. atrophaeus* strain HM03 suppressed the conidial germination of *C. gloeosporioides* and *C. acutatum*. This antagonistic effect might be due to the secretion of certain antifungal metabolites by *Bacillus* spp.^30,37^. This finding is in accordance with a previous report by Ramarathnam et al.^38^, who reported that *Bacillus* spp. played a role in producing antibiotic compounds to protect canola and wheat from fungal pathogens. Similarly, in a study by Cazorla et al*.*^39^, four *B. subtilis* strains isolated from avocado rhizoplane exhibited in vitro antagonistic activity against soil-borne phytopathogenic fungi using a dual culture plate assay. This report agrees with our previous study^8^, and another report by Zheng et al*.*^40^.

However, on the other hand, the development of fungicide resistance in pathogenic fungal isolates is one of the rising threats in plant disease management strategies^41^. To select the fungicide-resistant isolate, a previous study by Ramdial et al*.*^42^ reported that two fungal species, *Fusarium incarnatum* and *Colletotrichum truncatum* which cause diseases in bell pepper, were selected among several isolates that had shown resistant to fungicides based on in vitro bioassays. Moreover, *Colletotrichum* spp., are reported to become resistant to benzimidazole fungicides after prolonged use, with ultimate selection for resistant isolates in the fungal population^43^. In this study, the microscopic findings indicated that the suppressive effects of bacterial suspensions and its CF might be due to antibiosis mechanisms (Fig. S7). The selected AK-0 strain affected both the conidia germ tube and mycelial morphology of *C. gloeosporioides* YCHH4, causing severe damage to the fungal hyphae. Structural deformations caused by *Bacillus* antagonists in phytopathogenic fungi have been attributed to proteins, volatile compounds^44^, or root exudates^45^, being involved in biocontrol interaction by a *B. amyloliquefaciens* strain on *C. gloeosporioides* mycelia^46^.

Our strain AK-0 showed further PGP ability by producing IAA (Fig. S8). In support of this, a recent study by Asari et al*.*^47^ reported that *B. amyloliquefaciens* UCMB5113 was found to secrete IAA constitutively, and this production was increased when bacteria were grown in the presence of root exudates and was further stimulated by tryptophan, which has been demonstrated to be an IAA precursor in *Bacillus*-related strains^48,49^. However, these results contradict the previous reports of several researchers^50–52^, where the PGP activity was due to a wide range of mechanisms, such as phosphate solubilization, siderophore production, and biological nitrogen fixation. The AK-0-treated red-pepper plants showed better PGP ability than did the non-treated control. This suggested that AK-0 harbors genes encoding tryptophan-dependent IAA biosynthesis affecting plant growth, which were identified and are shown in Table S9.

To increase the bioefficacy and durability of the strain AK-0 for a longer period, the strain was formulated by mixing it with certain chemical ingredients to create AK-0PF. Nowadays, bacteria-based product formulations have received significant attention as a competitor for chemical fungicides to control plant diseases for a safe environment^53^. The AK-0PF was evaluated for the suppression of apple bitter rot on matured apples (Fig. 1A,B) and in vitro conidial germination of *C. gloeosporioides* (Fig. 1C). Previously, the microscopic observations by Pane and Zaccardelli^54^ found *Bacillus* strains to have a suppression effect on the spore germination of *Alternaria alternata* by *Bacillus* strains. Similar results have been displayed on the antagonistic effect of *B. amyloliquefaciens* to suppress wilt (*Fusarium oxysporum*) in banana and cucumber^55,56^.

Furthermore, AK-0PF has been identified as a potential BCA to control bitter rot in apple orchards under field conditions in Korea. There was no change in the effectiveness of AK-0PF compared to that in the AK-0 cell suspensions treatment in terms of controlling bitter rot on apple orchards. *B. subtilis* species are being commercialized in the market as biocontrol products against various plant diseases, including blight of apple, under pre- or post-harvest conditions^57,58^. *B. licheniformis* has previously been shown to be effective against anthracnose of mango fruits when it is applied as a liquid or powder formulations^59^. In contrast, a study by Ing et al*.*^60^ reported that the non-formulated chitosan exhibited antagonistic activity against fungal pathogens, including *Fusarium solani*, and *Aspergillus niger*, and *Colletotrichum musae*^61^. The combination of antagonists with other antimicrobial compounds; salts, such as bicarbonates; and natural compounds, such as chitosan, have improved the yield of BCA^62^. The general objective of most of the research groups related to biocontrol is to develop a product with a commercial purpose, but only a few are commercially available^63^. AK-0PF treatment further contributed to slightly increasing the chlorophyll content in matured leaves, while there was no change in the chlorophyll content in the non-treated control. A recent study determined that chickpea seedlings were treated with PGPR *Bacillus* spp. significantly enhanced the chlorophyll content by increasing photosynthetic rate^64^, resulting in the improvement of plant growth.

Our study further reports the whole genome sequence of the strain AK-0, which consists of a 3.97 Mb chromosome. The AK-0 genome was compared with another *Bacillus* spp. (Table S6). In particular, the biocontrol-related genes and gene clusters involved in the antibiotic could result in the differences in biocontrol targets and efficacy between AK-0 and other *Bacillus* strains. These results suggest that all other strains could prevent disease by the genes involved in the synthesis of secondary metabolites; these belong to the iturin family and exhibit strong antifungal activities against various fungal pathogens^65^. *Bacillus* species have been found to produce iturins extensively^66^, which explains why the biocontrol activity of PGPR can be mediated by their secondary metabolites^67^. This might have contributed to providing antagonistic activity against *C. gloeosporioides*. The whole-genome comparisons revealed that our strain AK-0 showed high similarity to *B. velezensis* FZB42 and *B. velezensis* DSM7, which is currently used as a BCA and biofertilizer^68^. Of the 3921 identified genes, 3403 were classified into different functional categories based on the designation of clusters of orthologous genes (COGs)^69^. The COG database was used to functionally categorize predicted proteins^70^, and we made a comparison of COG categories among the twenty strains. The COGs of the twenty strains showed highly similar distributions, suggesting that these strains have comparable biological niches. A total of ten putative secondary metabolite biosynthetic gene clusters have been identified in the AK-0 genome. Some of them were implicated in antibiosis^71^, and some of them were assigned COG dedicated to the transport and metabolism of amino acids, carbohydrates, lipids transport and metabolism, and catabolism of secondary metabolites^72^. These functions are essentials for a biocontrol agent for antagonism against various phytopathogens. The AK-0 genome was investigated for the presence of BGCs of potential interest, as identified by antiSMASH. The most shared clusters were those showing similarities to the known clusters which produce secondary metabolites, which play an important role in the suppression of pathogens by induced systemic resistance^73^.

Surfactin was reported to have a broad spectrum of antibacterial activity, and to significantly inhibit bacterial diseases in plants^74^. Plantazolicin, originally obtained from *B. velezensis* strain FZB42, displaying antibacterial activity against pathogenic bacteria such as *B. anthracis*^75^. Similarly, bacilysin, against the genus *Xanthomonas*^5^, and difficidin from a *B. subtilis* strain displayed broad spectra of antibacterial activities^76^. The secondary metabolites of AK-0 were identified from COG function annotations and compared to those of other *Bacillus* species. The secondary metabolite biosynthetic genes of AK-0 results were consistent. Table S10 shows the differences in the production of secondary metabolites among various *Bacillus* species, represented in different colored circles. The compound 2,3-butandiol (2,3-BD), which was identified from the AK-0, is involved in antimicrobial activity, and the genes are shown in Table S11. A total of 14 genes were involved in the 2,3-BD biosynthesis pathway from AK-0, with a fungal inhibitory effect due to the involvement of the above-mentioned genes. While, the group of NRPS clusters comprises surfactin, a representative of the iturin group (iturin A), a representative of the plipastatin group (fengycin), the siderophore bacillibactin, and the antibacterial bacilysin^77^. On the other hand, the genome also harbors three PKS gene clusters, which encode the antibacterial polyketides bacillaene, difficidin, and macrolactin.

In conclusion, the strain AK-0 isolated from the ginseng rhizosphere was identified and shown to exhibit significant activity against several fungal pathogens, including *C. gloeosporioides*, which causes bitter rot in apple. The strain was identified as *B. velezensis* AK-0 based on the whole-genome sequence analysis. The strain exhibits in vitro antagonistic activity and suppression of the conidial germination, and in planta disease control of bitter rot. AK-0PF also has been shown to control apple bitter rot without having any difference in the control effect by AK-0 cell suspensions under field conditions at two different apple orchards in Gyeongbuk Province, Korea. Whole-genome sequencing revealed that the core genome of AK-0 was very similar to those of various *B. velezensis* strains. The differences between the various *B. velezensis* strains in terms of the control targets and efficacies might be attributable to the variations in the genes or gene clusters responsible for the biocontrol mechanism. Moreover, several CLP products of this strain were determined through the analysis of secondary metabolite BGCs using antiSMASH software. AK-0 showed the potential for several biosynthetic compounds by PCR, which could play an important role in the disease control of apple bitter rot caused by *C. gloeosporioides* through a mechanism of antibiosis. Our results indicated that AK-0 might be a promising BCA to control phytopathogens in an eco-friendly manner. Future studies will apply proteomics and transcriptomics methods to investigate the signaling pathways involved in the antagonistic effects of secondary metabolites, including fengycin, and bacillomycin, against pathogenic fungi.

## Materials and methods

### Disease occurrences of anthracnose on apple orchards in the northern Gyeongbuk Province in Korea

The survey of the disease occurrence of apple bitter rot during from 2017 to 2019 is provided in the SI.

### General genomic features of *Bacillus velezensis* AK-0

Bacterial genomic DNA was extracted using a bacterial genomic DNA kit, and a description of whole-genome sequencing is provided in the SI. The complete sequence has been deposited in NCBI (accession number CP047119).

### Genome components and genome annotation

A detailed description of this method is provided in the SI.

### Genome mining of secondary metabolite gene clusters with biocontrol functions

The antiSMASH serves as a comprehensive resource for the automatic genomic identification and analysis of BGCs of any type, facilitating the rapid genome mining of microbial isolates^78^. Thereby, secondary metabolite BGCs in the AK-0 genome were mined using antiSMASH 4.1.0, and further aligned using NCBI Blast searches against different databases.

### Detection of genes for secondary metabolites from *B. velezensis* AK-0 using qPCR

Total RNAs from AK-0 cells were isolated from 2-day-old cultures grown in brain heart infusion (BHI) broth using the RNeasy mini kit with on-column DNase I treatment according to the manufacturer’s instructions (Qiagen Inc., Hilden, Germany). A detailed method for the detection of genes for secondary metabolites from AK-0 is provided in the SI.

### Plant growth-promoting effects by determination of IAA quantification and germination of red-pepper seeds

The IAA quantification assay was performed using the method described by Meza et al*.*^79^. For the quantitative determination of IAA, the colorimetric Salkowski’s assay was performed^80^. A detailed method is provided in the SI.

### Additional experiments

Additional experiments, including the isolation of pathogenic *C. gloeosporioides* fungus from the apple orchards and microscopic observation, preparation of fungal pathogen inocula, inhibition of spore germination of *C. gloeosporioides* by treatment with AK-0 cell suspensions, in vitro antagonistic activity assay, disease suppression of bitter rot caused by *C. gloeosporioides* APEC18-004 using AK-0 cell suspensions on harvested apples, selection of fungicide-resistant *C. gloeosporioides*, in vitro assay on the spore germination of fungicide-resistant *C. gloeosporioides* using AK-0 cell suspensions and CF, preparation of AK-0PF, *in planta* and in vitro assays on the suppression of apple bitter rot and conidial germination of *C. gloeosporioides* by treatment with AK-0PF, field evaluation of the application of AK-0PF and agricultural chemical fungicides on the disease suppression of apple bitter rot, and determination of chlorophyll content, disease occurrences of bitter rot on apple orchards in the northern Gyeongbuk Province in Korea, General genomic features of *B. velezensis* AK-0**,** whole-genome (PacBio data) analysis, comparative genomics, calculation of pan-genome orthologous groups are described in detail in the SI.

### Statistical analysis

The data were subjected to analysis of variance (ANOVA) using SAS JMP software ver. 3. (SAS, 1995)^81^. Significant differences between treatment means were determined using the least significant difference (LSD) at *P* < 0.05. All experiments were carried out at least two times. For each experiment, the data were analyzed separately. The results of one representative experiment were shown.

## Supplementary Information

Supplementary Information.
