# Supplementary material for: Characterization of Bacillus velezensis AK-0 as a biocontrol agent against apple bitter rot caused by Colletotrichum gloeosporioides
Source: Sci Rep. 2021 Jan 12;11:626. doi: 10.1038/s41598-020-80231-2 (PMC7804190; doi:10.1038/s41598-020-80231-2)
Supplement: Supplementary file 1 — Supplementary Information. [file 41598_2020_80231_MOESM1_ESM.docx]

**Characterization of *Bacillus velezensis* AK-0 as a biocontrol agent against apple bitter rot caused by *Colletotrichum gloeosporioides***

**Running title:** Characterization of *Bacillus velezensis* AK-0, an effective biocontrol agent

Young Soo Kim^1^, Younmi Lee^1^, Wonsu Cheon^1^, Jungwook Park^2^, Hyeok-Tae Kwon^1^, Kotnala Balaraju^3^, Jungyeon Kim^1^, Yeo Jun Yoon^4^, Yongho Jeon^1^*

^1^Department of Plant Medicals, Andong National University, Andong 36729, Republic of Korea

^2^Department of Microbiology, Pusan National University, Pusan 46241, Republic of Korea

^3^Agricultural Science & Technology Research Institute, Andong National University, Andong 36729, Republic of Korea

^4^Research Department, KOREABIO Co., Ltd. 18514, Republic of Korea

*Corresponding author: Yongho Jeon

E-mail: [yongbac@andong.ac.kr](mailto:yongbac@andong.ac.kr)

Tel: +82-54-820-5507

Fax: +82-54-820-6320

**Supplementary information (SI)**

**Materials and methods**

**Isolation of pathogenic *C. gloeosporioides* fungus from the apple orchards and microscopic observation.** Symptomatic fruits for the isolation of bitter rot were collected from the apple orchards. Small sections (5 × 5 mm^2^) were excised from the margins of the lesions on the fruits. These sections were surface sterilized with 1% sodium hypochlorite (NaOCl) solution for 1 min and 70% ethanol for 30 s and were then rinsed twice with SDW. After sterilization, the tissues were dried on sterile filter paper. Bitter rot tissues were placed onto PDA (Difco, USA) plates, and incubated at 27 °C for seven days in the dark. Conidia from the mycelial growth of the fungi were mounted on glass slides in SDW and observed under a ProgRes SpeedXT ^core^ 3 Imager microscope using a differential interference contrast illumination.

**Preparation of fungal pathogen inocula.** The conidial suspensions (100 µl) were spread onto PDA plates and incubated at 25 °C for seven days. The conidia were harvested by pouring SDW onto the PDA plate with pathogenic fungi and scraping. The resulting suspensions were filtered through a double layer of cheesecloth. The concentration of spore suspensions was adjusted to 10^5^ conidia/mL using a hemocytometer before application.

**Inhibition of spore germination of *C. gloeosporioides* by treatment with AK-0 cell suspensions.** The conidia from seven-day-old cultures were collected from the PDA plates with SDW and adjusted to 10^5^ spores/mL using a hemocytometer. The conidia germination and appressorium formations from *C. gloeosporioides* were tested on a cover glass surface treated with AK-0 bacterial suspensions using a previously described method^1,2^. Briefly, conidia from the cultures grown on PDA plates for seven days were harvested and washed with ice-cold SDW twice. The conidia suspension (10 µL) was dropped onto a cellophane membrane that was attached to the glass slide. The conidial germination and formation of appressorium and primary hyphae in the AK-0 treatment were assessed during the incubation at 25 °C and at different durations (8, 16, 24, 32, 40, and 48 h) in the Petri-dishes containing moist paper. At least 50 measurements per structure were measured with a ProgRes SpeedXT ^core^ 3 Imager microscope using a differential interference contrast illumination.

***In vitro* antagonistic activity assay.** *In* *vitro* antagonistic activity was performed using a dual culture plate assay^3^ to screen the rhizobacterium against the fungal pathogen *C. gloeosporioides*. The mycelial disc of each pathogen (measuring 8 mm) was placed onto the center of the PDA plates (90 mm diameter) and the bacterial suspensions were placed in the periphery of the plates, leaving a 1.0 cm gap from the plate rim. The antagonistic activity against fungal pathogens was measured as a zone of inhibition after incubating the plates for seven days at 25 °C. Each treatment contained five replications, and the assay was performed three times; the results of one of the three experiments are shown here.

**Disease suppression of bitter rot caused by *C. gloeosporioides* APEC18-004 using AK-0 cell suspensions on harvested apples.** Apple fruits of similar size were selected for our study. The fruits were surface sterilized with 70% ethanol followed by 2% NaOCl for 5 min, and then washed three times with SDW and air-dried in a clean bench. Surface-sterilized apple fruits were wounded by piercing them 1 to 2 mm deep with a sterile pin, and then treated with 10 µL of *B. velezensis* AK-0 bacterial suspensions (10^8^ cfu/mL) and allowed to dry for 10 min. The spore suspensions (10 µL) of the pathogenic fungi were inoculated onto the wounds of the apple fruits. The diameter of the symptoms was observed 6–11 days after incubation at 25 °C and compared to the non-treated control. For pre-treatment, the apples were treated with AK-0 suspensions (10^8^ cfu/mL) and incubated at 25 °C for 24 h, followed by challenge inoculation with fungal spore suspensions (10^5^ spores/mL), and then incubated in moist conditions at 25 °C for five days. The apples were observed for diseased lesions after incubating them at 25 °C without moisture conditions. For post-treatment, the apples were inoculated with fungal spores (10^5^ spores/mL) and incubated at 25 °C for 24 h, followed by treatment with AK-0 bacterial suspensions (10^8^ cfu/mL) by the spray method. Each treatment consisted of 8 replicates (fruits), and each experiment was performed two times. Control value (%) was calculated as follows: (No. of diseased lesions in non-treated control – No. of diseased lesions in treated sample)/No. of diseased lesions in non-treated control × 100.

**Selection of fungicide-resistant *C. gloeosporioides.*** The chemical fungicides, pyraclostrobin (335 ppm) and tebuconazole (1000 ppm) dissolved in SDW were mixed in autoclaved PDA (cooled to around 50 °C). Before solidification of the medium, the PDA plus fungicide was added to the Petri-plates. The control plates contained PDA without fungicides. All the fungal pathogenic strains of *C. gloeosporioides* were cultured onto PDA plates for ten days. For each of the 19 fungal pathogenic strains, a mycelium plug (8 mm diameter) was transferred from the leading edge of an actively growing colony onto PDA plates amended with or without fungicides. The plates were incubated at 25 °C in darkness for three days. When the colony in the control plates (without fungicide) had covered 75% of the agar surface, the diameter of all colonies of the microorganism was measured. The percentage of inhibition of radial growth was calculated. The experiment was performed two times, with three replicates for each fungal isolate. Percentage (%) inhibition of mycelial growth rate was calculated as follows: Control inhibition zone (mm) - Average inhibition zone of the sample (mm)/Control inhibition zone (mm)×100.

***In vitro* assay on spore germination of fungicide-resistant *C. gloeosporioides* using AK-0 cell suspensions and culture filtrate (CF).** The conidia were harvested as per the above method. Conidia germination and appressorium formations from *C. gloeosporioides* were tested on a cover glass surface in AK-0 bacterial suspensions and CF treatment using the method described previously by Kim *et al*. (1998)^1^. Conidia suspensions (10 µL) were dropped onto the cellophane membrane that was attached to the glass slide. Conidial germination and the formation of the appressorium and primary hyphae in AK-0 cell suspensions (10^6^, or 10^7^, or 10^8^ cfu/mL) and CF treatment were assessed during the incubation at 4 and 24 h at 25 °C in Petri dishes containing moist paper. Later, the observations were made using a microscope (ProgRes SpeedXT ^core^ 3 Imager with a differential interference contrast illumination).

**AK-0 product formulation (AK-0PF).** To further evaluate the efficacy of the biocontrol agent AK-0 in the field conditions, the AK-0 was formulated with various compositions to make it commercialized and increase the storage capacity in a liquid form. Before testing the AK-0PF under field conditions, it was tested on apple fruits for disease suppression of bitter rot and conidial germination. The AK-0 product formulation is as follows: bacterial suspensions were inoculated into KOREABIO Agricultural Medium (KAM; glucose 5.45 g, MSG 3.25 g, yeast extract 0.67 g, KH_2_PO_4_ 0.27 g, CaCl_2_ 0.27 g, FeSO_4_ 0.005 g, MgSO_4_ 0.08 g, MnSO_4_ 0.005 g, CuSO_4_ 0.003 g, and ZnSO_4_ 0.003 g per liter) and incubated for 48 h at 32 ℃ and 150 rpm, in a fermentation tank under controlled conditions. Considering the KAM as 85%, the other 15% was formed from other ingredients, such as oregano oil (5%), rosin (1%), the surfactants polyalkyleneoxide modified heptamethyltrisiloxane (4%), the moisturizer Na-PCA (0.5%), the sunscreen Lowilite-62 (1%), and the stabilizator methyl oleate (4%) should be added.

***In planta* and *in vitro* assay on the suppression of apple bitter rot and conidial germination of *C. gloeosporioides* by treatment with AK-0 product formulation (AK-0PF).** AK-0PF was sprayed onto the apples, allowed to dry for 1 h, and then challenge inoculated with fungal spore suspensions using the spray method. The disease severity (%) was recorded three days after incubation at 25 °C and compared to the non-treated control (water). The conidia germination and number of appressoria formed by treatment with AK-0PF was recorded. For microscopic observations, the germination and appressoria were induced to develop on the surface of hydrophobic glass plates *in vitro* after 48 h. At least 50 measurements per structure were measured with a ProgRes SpeedXT ^core^ 3 Imager microscope using a differential interference contrast illumination. For scanning electron microscopy (SEM) analysis, the tissues were prepared following the method of Ge and Guest (2011)^4^ with minor modifications. Samples were collected at 0 and 48 h after challenge inoculation. Pathogenic conidial suspensions treated with AK-0PF were incubated for a different duration (0 – 48 h). Samples were dried for 30 min at 45 °C. The dried samples were directly subjected to gold coating in an ion coater. Thus, prepared specimens were observed using an SEM (Hitachi S-2500C; Jeol Co. Japan) at 10, 15, and 20 kV.

**Field evaluation of the application of AK-0PF and agricultural chemical fungicide on disease suppression of apple bitter rot.** AK-0PF and chemical fungicide (Tebuconazole) were applied to the apple orchards at two different locations (Yecheon and Mungyeong) in Gyeongbuk Province, Korea. Plants treated with water spray served as a non-treated control. The treatments were categorized into five groups. Control: plant treated with water, ACF: plants treated with agricultural chemical fungicides (Table S1) with the dosage as per the instructions given by the manufacturer, TBWP: plants treated with tebuconazole 25% wettable powder (WP), AK-0PF: Plants treated with AK-0 product formulation, and AK-0PF/TBWP: plants treated with AK-0PF or tebuconazole as an alternative spray every week. All the treatments were given as a foliar spray from early July 2019 to late October 2019 at two-week intervals in 100-fold dilution with 50 L per three apple trees. Each treatment contained three replicates (plants) in the experiment. After 15 days, the diseased fruits were harvested, and the size of the lesions were measured and disease incidence (%) was calculated from disease ratings, and control values were shown. Control value (%) was calculated as follows: (No. of diseased lesions in non-treated control – No. of diseased lesions in treated sample)/No. of diseased lesions in non-treated control × 100.

**Determination of chlorophyll content.** The chlorophyll content of the apple leaves was measured using the method described by Uddling *et al*. (2007)^5^. After the application of AK-0PF and chemical fungicides on the apple orchards at two different locations, the chlorophyll content was measured from the leaves of the one-year-old branch using a SPAD-502 meter. The chlorophyll content was measured from 20 leaves per tree with three replications in every two weeks.

**Disease occurrences of bitter rot on apple orchards in the northern Gyeongbuk Province in Korea.** For two years (2018–2019), the disease occurrence of bitter rot caused by *C.* *gloeosporioides* in apple orchards has been investigated in two different locations (Mungyeong and Yecheon) in northern Gyeongbuk Province, Korea. The disease occurrence was investigated in ‘Fuji’ apple cultivar from the orchards during the months of late June to early October at 15-day intervals (Table S2). For the disease survey analysis, 25 trees were randomly selected, and the rate of diseased fruits was recorded for their severities based on the disease index of each plant. The percentage of disease incidence for bitter rot in the fruits was recorded. The diseases were identified based on their diagnostic symptoms, following the guidebook by Choi *et al*. (2012)^6^.

**General genomic features of** ***B. velezensis* AK-0.** Bacterial genomic DNA was extracted using a bacterial genomic DNA kit, and whole-genome sequencing was performed using a PacBio RSII single-molecule real-time sequencing technique with a 20-kb SMRTbell library at Biomarker (Macrogen, Korea). The whole-genome sequencing was performed using a PacBio RSII (Pacific Biosciences Inc.) and the Illumina HiSeq X-Ten (Macrogen Inc.) platforms. A total of 136,625 subreads (N_50_ value of 12,509 bp) and 1,233,875,893 subread base pairs with 272× coverage were generated, and these subreads were assembled using the RS hierarchical genome assembly process (HGAP) (v3.0) and a single-molecule real-time (SMRT) portal (v2.3) *de novo* assembler. Gene annotation was performed using the NCBI Prokaryotic Genomes Automatic Annotation Pipeline (PGAAP) with the best-placed reference protein set GeneMarkS2 (v4.7) and the RAST server (<http://rast.nmpdr.org/>). The identified genes that were potentially involved in the biosynthesis of secondary metabolites for suppressing plant pathogenic fungi were found to be related to NRPS and PKS using antiSMASH 3.0^7^.

**Whole Genome (PacBio data) analysis.** The genome of AK-0 strain was constructed de novo using Pacbio sequencing data. Sequencing analysis was performed in Macrogen, Inc. PacBio sequencing data were assembled with PacBio SMRT Analysis 2.3.0 using the HGAP2 protocol (Pacific Biosciences, USA). Resulting contigs from PacBio sequencing data were circularized using Circlator 1.4.0 (Sanger institute). Gene-finding and functional annotation pipeline of whole genome assembles used in EzBioCloud genome database. Protein-coding sequences (CDSs) were predicted by Prodigal 2.6.2^8^. Genes coding for tRNA were searched using tRNAscan-SE 1.3.1^9^. The rRNA and other non-coding RNAs were searched by a covariance model search with Rfam 12.0 database^10^. The CDSs were classified into groups based on their roles, with reference to orthologous groups (EggNOG 4.5; http://eggnogdb.embl.de)^11^.

**Comparative genomics.** For comparative genomics, the genome sequences of closely related AK-0 strains were obtained from the EzBioCloud database^12^. The sequence of genomes were obtained from *Bacillus velezensis* strain QST713, CFSAN034339, UMAF6639, UCMB5036, AS43.3, UCMB5113, TrigoCor1448, FZB42, G341, UCMB5033, LS69, SQR9, YAU B9601-Y2, JS25R, NAU-B3, and IT-45, *Bacillus amyloliquefaciens* strain DSM 7 and TA208, and *Bacillus subtilis* subsp. *Subtilis* strain NCIB 3610 and 168. After a selection of genomes, a comparative genomic analysis was conducted using ChunLab’s comparative genomics tool (https://www.ezbiocloud.net/contents/cg). To understand the phylogenetic relationships of the analyzed strains, OrthoANI values were calculated, and UPGMA dendrograms were generated using the Orthologous ANI Tool (OAT) of ChunLab^13^.

**Calculation of pan-genome orthologous groups.** Pan-genome orthologous groups (POGs) were determined by a combined reciprocal best hit (RBH) method using uBLAST with an e-value threshold of 1 × 10^−614^ and an open reading frame (ORF)-independent method using nucleotide sequences with cutoff values of at least 70% of gene coverage^15^. After the initial grouping, partial genes that are grouped out due to its short sequence length are targeted for clustering analysis against the POGs using UCLUST (≥95% identity). A Venn diagram was drawn using jvenn^16^ with the calculated POGs.

**Genome components and genome annotation.** Coding DNA sequence (CDS) prediction was performed using Glimmer 3.02^17^. A circular map of the genome was obtained using Circos version 0.64^18^. Genomic islands (GIs) were predicted using the GI prediction method in IslandViewer 4^19^. The tRNA and rRNA were predicted using the tRNAscan-SEv1.3.1^20^ and barrnap 0.7 software, respectively. Clustered regularly interspaced short palindromic repeat sequences (CRISPRs) were found using CRISPRFinder^21^. Functional annotation was based on BLASTP searches (BLAST 2.2.28+) against the NCBI non-redundant (NR), gene database, string databases. Based on the string database, a BLASTP comparison was used to perform the Clusters of Orthologous Groups of proteins (COG) annotation, according to which the protein function could be classified^22^. The BLAST algorithm was used to compare the predicted genes with the Kyoto Encyclopedia of Genes and Genomes (KEGG) database, and the corresponding genes involved in specific biological pathways could be obtained according to the KEGG Orthology (KO) numbers obtained from the alignment. GO was annotated with blast2go^23^. To further characterize the metabolic changes and the metabolic pathways involved, the differentiated metabolites were first annotated with KEGG (<http://www.genome.jp/kegg/>).

**Detection of transcripts for secondary metabolites from *B. velezensis* AK-0 using qPCR.** The total RNAs from AK-0 cells were isolated from two-day-old cultures grown in BHI broth using the RNeasy mini kit with On-Column DNase I treatment according to the manufacturer’s instructions (Qiagen Inc., Hilden, Germany). The cDNA was generated using an iScript cDNA synthesis kit (Bio-Rad, Hercules, CA, USA) following the manufacturer’s protocol, with random hexamer primers and the DNase-treated total RNA as a template. Nine genes of secondary metabolites were selected for validation using real-time PCR. Primer sets were designed using Web Tools (Integrated DNA Technologies), and all the primers used are listed in Table S3. qRT-PCR was carried out using a CFX Connect Real-Time PCR Detection System (Bio-Rad), with 16S rRNA as a reference gene. Each reaction contained 10 ng of cDNA, 5 pmol each of forward and reverse primers, and SsoAdvanced SYBR Green Supermix (Bio-Rad). The conditions for thermal cycling were as follows: denaturation at 95 ℃ for 3 min for polymerase activation, followed by 40 cycles at 95 ℃ for 10 s and 60 ℃ for 30 s. After the last reaction cycle, melting curves were obtained through a temperature ramp from 65 ℃ to 95 ℃, with 0.5 ℃/s increments, to exclude nonspecific products. Each PCR run included a “no template” control sample, and all tests were performed in triplicate. Cycle threshold (Ct) values, relative to the control sample, were used to calculate ΔΔCt values (the difference between ΔCt values, calculated from the difference between the Ct of the target and the reference gene) for each sample. The constitutively expressed 16S ribosomal RNA gene was used for data normalization^24^.

**Plant growth-promoting effects by determination of IAA quantification and germination of red-pepper seeds.** An indole-3-acetic acid (IAA) quantification assay was performed using the method described by Meza *et al*. (2015)^25^. For the quantitative determination of IAA, the colorimetric Salkowski’s assay was performed^26^. IAA was quantified by integrating the areas under peaks with authentic IAA (Sigma) as the standard. For the germination assay, red-pepper (*Capsicum annuum* L. cv. ‘Hanbyul’) seeds were used for the growth-promoting assay in this study. Bacterial suspensions to be used for the treatment were prepared by inoculating BHI plates with AK-0 colonies and incubating them at 28 °C for 48 h. The suspensions were collected in SDW and adjusted to a final concentration of 1 × 10^6^ CFU/ml. The seeds were placed onto the germination trays (6 × 8 holes) containing soilless Flora Guard (TKS 2 INSTANT, Kultur substrate) for germination. Two-week-old seedlings were soil drenched with 20 mL of bacterial suspension of AK-0 or SDW (control). The plant lengths of the germinated seedlings were measured for two weeks after incubation at 28 ℃ under greenhouse conditions. The experiment was performed twice with 36 replicates (seedlings) in each treatment.

**References**

1. Kim, Y. K., Li, D. & Kolattukudy, P. E. Induction of Ca^2+^-calmodulin signaling by hard-surface contact primes *Colletotrichum gloeosporioides* conidia to germinate and form appressoria. *J Bacteriol* **180,** 5144–5150 (1998).
2. Flaishman, M. A., Hwang, C. H. & Kolattukudy, P. E. Involvement of protein phosphorylation in the induction of appressorium formation in *Colletotrichum gloeosporioides* by its host surface wax and ethylene. *Physiol Mol Plant Pathol* **47,** 103–17 (1995).
3. Fokkema, N. J. Fungal antagonism in the phylosphere. *Ann Appl Biol* **89,** 115–117 (1978).
4. Ge, Y. H. & Guest, D. Light and scanning electron microscopy studies on the infection process of melon leaves by *Colletotrichum lagenarium*. *Physiol Mol Plant Pathol* **76,** 67–74 (2011).
5. Uddling, J., Gelang-Alfredsson, J., Piikki, K. & Pleijel, H. Evaluating the relationship between leaf chlorophyll concentration and SPAD-502 chlorophyll meter readings. *Photosynthesis Res* **91,** 37–46 (2007).
6. Choi *et al*. An illustrated guide to apple pest in Korea. RDA, Suwon, Korea (2012).
7. Weber, T. *et al*. antiSMASH 3: 0 – a comprehensive resource for the genome mining of biosynthetic gene clusters. *Nucleic Acids Res* **43,** W237–W243 (2015).
8. Hyatt, D. *et al*. Prodigal: prokaryotic gene recognition and translation initiation site identification. *BMC Bioinformatics* **11,** 119 (2010).
9. Schattner, P., Brooks, A. N. & Lowe TM. The tRNAscan-SE, snoscan and snoGPS web servers for the detection of tRNAs and snoRNAs. Nucleic Acids Res **33,** 686–689 (2005).
10. Nawrocki, E. P. & Eddy, S. R. Infernal 1.1: 100-fold faster RNA homology searches. *Bioinformatics* **29,** 2933–2935 (2013).
11. Powell, S. *et al*. eggNOG v4.0: nested orthology inference across 3686 organisms. *Nucleic Acids Res* **42,** D231–239 (2014).
12. Yoon, S. H. *et al*. Introducing EzBioCloud: A taxonomically united database of 16S rRNA gene sequences and whole-genome assemblies. *Int J Syst Evol Microbiol* **67,** 1613–1617 (2017).
13. Lee, I., Kim, Y. O., Park, S. C. & Chun, J. OrthoANI: An improved algorithm and software for calculating average nucleotide identity. *Int J Syst Evol Microbiol* **66,** 1100–1103 (2016).
14. Ward, N. & Moreno-Hagelsieb, G. Quickly finding orthologs as reciprocal best hits with BLAT, LAST, and UBLAST: How much do we miss? *PLoS One* **9,** 2101850 (2014).
15. Chun, J. *et al*. Comparative genomics reveals mechanism for short-term and long-term clonal transitions in pandemic *Vibrio cholerae*. *Proc Natl Acad Sci USA* **106,** 15442–15447 (2009).
16. Bardou, P., Mariette, J., Escudié, F., Djemiel, C. & Klopp, C. Jvenn: An interactive Venn diagram viewer. *BMC Bioinformatics* **15,** 293 (2014).
17. Delcher, A. L., Harmon, D., Kasif, S., White, O. & Salzberg, S. L. Improved microbial gene identification with GLIMMER. *Nucleic Acids Res* **27,** 4636–4641 (1999).

1. Krzywinski, M. *et al*. Circos: an information aesthetic for comparative genomics. *Genome Res* **19,** 1639–1645 (2009).
2. Dhillon, B. K. et al. IslandViewer 3: more flexible, interactive genomic island discovery, visualization and analysis. *Nucleic Acids Res* **43,** W104–W108 (2015).
3. Lowe, T. M. & Eddy, S. R. tRNAscan-SE: a program for improved detection of transfer RNA genes in genomic sequence. *Nucleic Acids Res* **25,** 955–964 (1997).
4. Grissa, I., Vergnaud, G. & Pourcel, C. CRISPRFinder: a web tool to identify clustered regularly interspaced short palindromic repeats. *Nucleic Acids Res* **35,** W52–W57 (2007).
5. Tatusov, R. L. *et al*. The COG database: new developments in phylogenetic classification of proteins from complete genomes. *Nucleic Acids Res* **29,** 22–28 (2001).

1. Conesa, A. et al. Blast2GO: a universal tool for annotation, visualization and analysis in functional genomics research. *Bioinformatics* **21,** 3674–3676 (2005).
2. Yu, Z., Guo, C. & Qiu, J. Precursor amino acids inhibits polymyxin E biosynthesis in *Paenibacillus polymyxa*, probably by affecting the expression of polymyxin E biosynthesis-associated gens. *BioMed Res Inter Article* ID 690830, 11 pages (2015).
3. Meza, B., de-Bashan, L. E., & Bashan, Y. Involvement of indole-3-acetic acid produced by *Azospirillum brasilense* in accumulating intracellular ammonium in *Chlorella vulgaris*. *Res Microbiol* **166,** 72–83 (2015).
4. Gordon, S. A. & Weber, R. P. Colorimetric estimation of indoleacetic acid. *Plant Physiol* **26,** 192–195 (1951).
5. Lee, I., Kim, Y. O., Park, S. C. & Chun, J. OrthoANI: An improved algorithm and software for calculating average nucleotide identity. *Int J Syst Evol Microbiol* **66,** 1100–1103 (2016).

**Supplementary Tables**

**Table S1.** Schemes of agricultural chemicals (fungicides and insecticides) use in control of bitter rot and pests in apple orchards in Korea.

| **S. No.** | **Date** | **Mungyeong** | | **Yecheon** | |
| --- | --- | --- | --- | --- | --- |
|  |  | **Fungicide** | **Insecticide** | **Fungicide** | **Insecticide** |
| 1 | 04-24 | Iminoctadine triacetate 25% | Acetamiprid 8% | Thiophanate-methyl 70% | Thiacloprid 10% |
| 2 | 05-08 | Fluquinconazole 10% | Chlorantraniliprole 4% | Mancozeb 65% | Chlorantraniliprole 5% |
| 3 | 05-21 | Difenoconazole 10% | Acetamiprid 2.5% + Etofenprox 8% | Metconazole 20% | Methoxyfenozide 4% |
| 4 | 06-03 | Kresoxim-methyl 44.2% | Methoxyfenozide 4% | Fluazinam 50% | Acetamiprid 2.5% |
| 5 | 06-17 | Dithianon 75% | Sulfoxaflor 7% | Kresoxim-methyl 44.2% | Imidacloprid 4% + Methoxyfenozide 8% |
| 6 | 07-01 | Fluazinam 50% | Cyfluthrin 5% | Dithianon 75% | Indoxacarb 1% + Teflubenzuron 2% |
| 7 | 07-15 | Trifloxystrobin 22% | Teflubenzuron 5% | Trifloxystrobin 22% | Etofenprox 8% + Methoxyfenozide 3.2% |
| 8 | 07-29 | Metconazole 20% | Alpha-cypermethrin 2% | Iminoctadine tris(albesilate) 20% | Teflubenzuron 5% |
| 9 | 08-12 | Chlorothalonil 75% | Tebufenozide 20% | Chlorothalonil 75% | Alpha-cypermethrin 2% |
| 10 | 08-26 | Azoxystrobin 10% | Novaluron 10% | Dithianon 11.4% + Pyraclostrobin 3.8% | Chlorpyrifos 20% |
| 11 | 09-09 | Tebuconazole 25% WP | Lufenuron 5% | Chlorothalonil 20% +Difenoconazole 4% | Novaluron 10% |
| 12 | 09-23 | Difenoconazole 3% + Iminoctadine triacetate 15% | Deltamethrin 1% | Difenoconazole 3% + Iminoctadine triacetate 15% | Deltamethrin 1% |

**Table S2.** Detailed information of the apple orchards for disease observations for two years (2018 – 2019)

| **Location** | | **Root stock** | **Cultivar** | **No. of fungicide spray/year** | **Age of the tree (year)** | **Cultivation periods (year)** |
| --- | --- | --- | --- | --- | --- | --- |
| **Mungyeong** | Jeomchon Jingok-ro 2 | M26 | Fuji | 10+α | 23 | 23 |
|  | Mungyeong-eup, Juheul-ro 176-21 | MM106 | Fuji | 12 | 25 | 28 |
|  | Sanbuk-myeon, Geumcheon-ro 744 | M26 | Fuji | 10+α | 18 | 21 |
|  | Jingok-ro 2, 22-5 | M9 | Fuji | 10+α | 15 | 30 |
| **Yecheon** | Eunpung-myeon, Eunsan-ro 98 | M9 | Fuji | 11+α | 14 | 21 |
|  | Eunpung-myeon, Eunsan-ro 42 | M9 | Fuji | 11+α | 15 | 18 |
|  | Hari-myeon, Dohyoja-ro 984-7 | M9 | Fuji | 12+α | 15 | 17 |
|  | Jibo-myeon, Manhwa-ro 2, 270 | M9 | Fuji | 11+α | 25 | 30 |

**Table S3.** Primers used for qPCR to gene expression of secondary metabolites in *Bacillus velezensis* AK-0

| **Antibiotic** | **Gene** | **Primers** | **Primer sequence (5’-3’)** | **PCR product size**  **(bp)** | **Melting temperature (Tm)** |
| --- | --- | --- | --- | --- | --- |
| Iturin | *ituD* | ituD-F | GCCATAGCTTAGGCGAATA | 199 | 53.6 |
|  |  | ituD-R | GGAAAGTCTTCCGTCGATAC |  | 54.8 |
| Iturin A | *ituA* | ituA-F | CAAGCCTCTGGCGTTATATG | 219 | 55.4 |
|  |  | ituA-R | GTCAGCACATGCTCGATAAA |  | 55.3 |
| Difficidin | *dfnA* | dfnA-F | CCCATTACCTATGCCGAAAG | 244 | 55 |
|  |  | dfnA-R | GATCCGTTCGATACTCAATCC |  | 55.1 |
| Bacilysin | *bacD* | bacD-F | TCGGTCGCGGTCATAAA | 195 | 54.9 |
|  |  | bacD-R | TGCCATCGGAGCGATAA |  | 54.6 |
| Baillomycin | *bmyA* | bmyA-F | TCCAACCCGACCTTATGA | 197 | 53.7 |
|  |  | bmyA-R | CAATTCCCGGTACGTTAGAC |  | 55 |
| Fengycin | *Fen* | fenC-F | CGGCTCCACTTTGTATATGG | 209 | 55.1 |
|  |  | fenC-R | GGACTTCCGCCAAGTAATC |  | 54.8 |
| Surfactin | *srfA* | srfAD-F | GGACACGGAACAAATCAAATG | 203 | 55.1 |
|  |  | srfAD-R | GGCTGAATGGCTGAGATG |  | 54.6 |
| Macrolactin | *mlnA* | mnlA-F | CTCGGTGAAATGGACTGAAA | 203 | 54.7 |
|  |  | mnlA-R | CCTTCTCTTTCTCGCTTTCC |  | 55.2 |

**Table S4.** Occurrence of disease severity of bitter rot on apple orchards in Gyeongbuk Province during 2018 and 2019

| **Location** | **Disease severity (%)^a^** | |
| --- | --- | --- |
|  | **2018** | **2019** |
| Jeomchon Jingok-ro 2 | 5.73±0.91 | 9.85±0.65 |
| Mungyeong-eup, Juheul-ro 176-21 | 6.54±2.4 | 6.48±1.25 |
| Sanbuk-myeon, Geumcheon-ro 744 | 4.7±1.03 | 3.04±0.87 |
| Jingok-ro 2, 22-5 | 5.21±1.12 | 2.5±0.77 |
| Eunpung-myeon, Eunsan-ro 98 | 5.54±0.59 | 9.78±0.65 |
| Eunpung-myeon, Eunsan-ro 42 | 3.65±2.23 | 4.19±0.24 |
| Hari-myeon, Dohyoja-ro 984-7 | 6.62±1.56 | 8.65±1.7 |
| Jibo-myeon, Manhwa-ro 2, 270 | 9.36±1.43 | 18.54±2.6 |
| Average | 5.92±1.41 | 7.88±1.09 |

^a^The disease severity of bitter rot on apple was recorded and evaluated by the disease index scale; 0 = no visible symptom, 1 = less than one fifth of fruits with visible symptom, 2 = less than two fifths of fruits with visible symptom, 3 = less than three fifths of fruits with visible symptom, 4 = less than four fifths of fruits with visible symptom, 5 = more than four fifths of fruits with visible symptom. Disease severity (%) = [∑ (disease index × the number of diseased fruits)/(the highest disease index × the number of plant rated)] × 100.

**Table S5.** Comparison of the morphological characteristics of the strain *Colletotrichum gloeosporioides* used in this study with those of previously reported *Colletotrichum* spp*.*

| **Species** | **Colony Color** | **Conidia** | | | **Appressoria** | | |
| --- | --- | --- | --- | --- | --- | --- | --- |
|  |  | **Length**  **(µm)** | **Width**  **(µm)** | **Shape** | **Length**  **(µm)** | **Width**  **(µm)** | **Shape** |
| APEC18-004 | White to light gray, thick cottony mycelia with orange conidial masses | 12.6–18.6 | 4.3–5.8 | Cylindrical, straight, apex obtuse | 9.2–17.5 | 4.5–11.3 | Clavate |
| *C. gloeosporioides* | Varied | 9–24 | 3–4.5 | Straight, obtuse at apex | 6–20 | 4–12 | Clavate to irregular |
| *C. acutatum* | White to pinkish grey or orange colored colony with slight mycelium | 8.5–10 | 4.5–6 | Fusiform, medianly constricted | 8.5–10 | 4.5–6 | Clavate or irregular |
| *C. capsici* | White to gray color with dark green center and cottony mycelium | 18–23 | 3.5–4 | Falcate, fusiform apices acute | 9–14 | 6.5–11.5 | Clavate to circular |
| *C. cocodes* | White mycelia | 16–22 | 3–4 | Fusiform, medianly constricted | 11–16.5 | 6–9.5 | Long clavate irregular |

**Table S6.** Comparative genomic analysis* of the *Bacillus* *velezensis* AK-0 with genomes of other *Bacillus* spp.

| **GenBank accession** | **Taxon name** | **Strain name** | **Source of isolation** | **Genome size (bp)** | **DNA G+C content (%)** | **No. of CDSs** | **No. of rRNA genes** | **No. of tRNA genes** |
| --- | --- | --- | --- | --- | --- | --- | --- | --- |
| CP047119 | *Bacillus velezensis* | AK-0 | Korean ginseng rhizosphere | 3,969,429 | 46.5 | 3,808 | 27 | 86 |
| GCA_003073255.1 | *Bacillus velezensis* | QST713 | Commercial product Serenade (Bayer) | 4,233,757 | 45.9 | 4,135 | 25 | 79 |
| GCA_001709055.1 | *Bacillus velezensis* | CFSAN034339 | Agricultural soil | 4,209,526 | 45.9 | 4,149 | 12 | 79 |
| GCA_001593765.1 | *Bacillus velezensis* | UMAF6639 | Unknown | 4,034,636 | 46.3 | 3,840 | 27 | 83 |
| GCA_000341875.1 | *Bacillus velezensis* | UCMB5036 | Cotton plant | 3,910,324 | 46.6 | 3,724 | 31 | 89 |
| GCA_000319475.1 | *Bacillus velezensis* | AS43.3 | Unknown | 3,961,368 | 46.6 | 3,808 | 31 | 89 |
| GCA_000455585.1 | *Bacillus velezensis* | UCMB5113 | Unknown | 3,889,532 | 46.7 | 3,698 | 31 | 89 |
| GCA_000583065.1 | *Bacillus velezensis* | TrigoCor1448 | Wheat plant | 3,957,904 | 46.5 | 3,779 | 24 | 77 |
| GCA_000015785.1 | *Bacillus velezensis* | FZB42 | Plant-pathogen infested soil and its organic material | 3,918,589 | 46.5 | 3,736 | 31 | 89 |
| GCA_001023595.1 | *Bacillus velezensis* | G341 | Four-year-old Korean ginseng roots | 4,009,746 | 46.5 | 3,861 | 30 | 95 |
| GCA_000455565.1 | *Bacillus velezensis* | UCMB5033 | Unknown | 4,071,167 | 46.2 | 3,934 | 30 | 86 |
| GCA_001687745.1 | *Bacillus velezensis* | LS69 | Rice field | 3,917,761 | 46.5 | 3,749 | 21 | 72 |
| GCA_000685725.1 | *Bacillus velezensis* | SQR9 | Unknown | 4,117,023 | 46.1 | 3,959 | 21 | 72 |
| GCA_000284395.1 | *Bacillus velezensis* | YAU B9601-Y2 | Unknown | 4,242,774 | 45.9 | 4,142 | 30 | 91 |
| GCA_000769555.1 | *Bacillus velezensis* | JS25R | Wheat | 4,014,440 | 46.4 | 3,818 | 21 | 83 |
| GCA_000493375.1 | *Bacillus velezensis* | NAU-B3 | Unknown | 4,204,608 | 46.0 | 4,099 | 30 | 92 |
| GCA_000242855.2 | *Bacillus velezensis* | IT-45 | Unknown | 3,936,866 | 46.6 | 3,796 | 30 | 95 |
| GCA_000196735.1 | *B. amyloliquefaciens* | DSM 7^T^ | Soil | 3,980,199 | 46.1 | 4,039 | 30 | 94 |
| GCA_000195515.1 | *B. amyloliquefaciens* | TA208 | Unknown | 3,937,511 | 45.8 | 4,000 | 19 | 70 |
| GCA_002055965.1 | *B. subtilis* subsp. *subtilis* | NCIB 3610^T^ | Unknown | 4,299,822 | 43.3 | 4,329 | 30 | 88 |
| GCA_000009045.1 | *B. subtilis* subsp. *subtilis* | 168 | Unknown | 4,215,606 | 43.5 | 4,220 | 30 | 86 |

*The comparative analysis data was analyzed using EzBioCloud Comparative Genomics Database by ChunLab, Inc. (<http://cg.ezbiocloud.net/>)

**Table S7.** Comparison on COG functional categories of four biocontrol strains

| **COG code** | **Description** | **AK-0** | **FZB42** | **CAU B946** | **M75** |
| --- | --- | --- | --- | --- | --- |
| C | Energy production and conversion | 198 | 169 | 167 | 181 |
| D | Cell cycle control, cell division, chromosome partitioning | 77 | 37 | 36 | 33 |
| E | Amino acid transport and metabolism | 339 | 275 | 286 | 287 |
| F | Nucleotide transport and metabolism | 92 | 82 | 81 | 81 |
| G | Carbohydrate transport and metabolism | 290 | 236 | 232 | 241 |
| H | Coenzyme transport and metabolism | 199 | 120 | 116 | 100 |
| I | Lipid transport and metabolism | 139 | 122 | 121 | 111 |
| J | Translation, ribosomal structure, and biogenesis | 241 | 155 | 156 | 156 |
| K | Transcription | 304 | 240 | 247 | 246 |
| L | Replication, recombination, and repair | 135 | 112 | 122 | 137 |
| M | Cell wall/membrane/envelope biogenesis | 247 | 174 | 183 | 188 |
| N | Cell motility | 72 | 61 | 62 | 48 |
| O | Posttranslational modification, protein turnover, chaperones | 141 | 97 | 100 | 98 |
| P | Inorganic ion transport and metabolism | 190 | 154 | 149 | 181 |
| Q | Secondary metabolites biosynthesis, transport, and catabolism | 117 | 64 | 65 | 87 |
| R | General function prediction only | 360 | 337 | 345 | 290 |
| S | Function unknown | 266 | 318 | 330 | 783 |
| T | Signal transduction mechanisms | 201 | 130 | 122 | 137 |

**Table S8.** Secondary metabolite biosynthetic gene clusters in *Bacillus velezensis* AK-0

| **Metabolite** | **Genes and gene clusters** | **Enzyme** | **Size**  **(kb)** | **Functions** | **Effect against** | **Identity**  **(%)** |
| --- | --- | --- | --- | --- | --- | --- |
|  |  | **Nonribosomal synthesis of lipopeptides (LP)** | | |  |  |
| Surfactin | srfABCD | NRPS | 26.1 | Biofilm, Induction of ISR | Fungi | 91 |
| Fengycin | fenABCDE | NRPS | 37.5 | Induction of ISR | Fungi | 100 |
| Bacillomycin | dmyCBAD | NRPS/PKS | 37.6 | Induction of ISR | Fungi | 100 |
| Bacillibactin | dhbABCDEF | NRPS | 12.5 | Siderophore production | Microbial competitors | 100 |
|  |  | **Nonribosomal synthesis of polyketides (PK)** | | |  |  |
| Difficidein | difAYXBCDEFGHIKLM | NRPS | 69.8 | Direct suppression | Bacteria | 100 |
| Bacillaene | baeBCDE, acpK, baeGHIJLMNRS | PKS/NRPS | 72.5 | Direct suppression | Bacteria | 100 |
| Macrolactin | mlnABCDEFGHI | NRPS | 54.8 | Direct suppression | Bacteria | 100 |
| Butirosin | kijA1-A8S4B1-B3C1-C5EDBAS5S3S2S1D1-D12 | PKS | 28.2 | Direct suppression | Antibacterial | 7 |
|  |  | **Nonribosomal synthesis of dipeptide antibiotics** | | |  |  |
| Bacilysin | bacABCDE, ywfG | NRPS | 5.9 | Direct suppression | Bacteria, Nematodes | 100 |
| Kijanimicin |  | Thiopeptide | 99.9 |  |  | 4 |

**Table S9.** Secondary metabolite biosynthetic genes related to Trp-dependent indole-3-acetic acid (IAA) in *Bacillus velezensis* AK-0

| **Gene** | **Product** |
| --- | --- |
| *dhaS* | Putative indole-3-acetaldehyde dehydrogenase |
| *ysnE* | Putative IAA acetyl-transferase |
| *yhcX* | Nitrilase |
| *trpE* | Anthranilate synthase |
| *trpD* | Anthranilate phosphoribosyltransferase |
| *alsD* | Alpha-acetolactate decarboxylase |
| *alsS* | Alpha-acetolactate decarboxylase |
| *alsR* | Alpha-acetolactate decarboxylase |
| *bdhA* | (R,R)-butanediol dehydrogenase BdhA |
| *dhaS* | Aldehyde dehydrogenase |

| **Species** | **Strain** | **Secondary metabolites** |
| --- | --- | --- |
| *Bacillus velezensis* | AK-0 |  |
|  | FZB42 |  |
|  | YAU B9601-Y2 |  |
|  | JS25R |  |
|  | NAU-B3 |  |
|  | SQR9 |  |
|  | UCMB5036 |  |
|  | UCMB5033 |  |
|  | UCMB5113 |  |
|  | TrigoCor1448 |  |
|  | AS43.3 |  |
| *Bacillus amyloliquefaciens* | KHG19 |  |
|  | IT-45 |  |
|  | LFB112 |  |
|  | L-S60 |  |
|  | DSM7 |  |
|  | LL3 |  |
|  | TA208 |  |
|  | XH7 |  |
| *Bacillus licheniformis* | DSM 13 ATCC 14580 |  |
| *Bacillus subtilis* | SR1 |  |
|  | GS 188 |  |
| *Bacillus siamensis* | XY18 |  |
|  | CKTC13613 |  |
| *Bacillus cereus* | ATCC 10876 |  |
|  | ATCC 4342 |  |
| *Bacillus anthracis* | 03BB102 |  |

**Table S10.** Secondary metabolite biosynthetic gene clusters of other *Bacillus* species from COG function annotation

Surfactin, Bacillaene, Bacillibactin, Bacillomycin/iturin, Difficidin, Fengycin, Macrolactin, Petrobactin, Zwittermycin, No gene

**Table S11.** Butanoate metabolism and 2,3-BD biosynthesis pathway

| Gene | Product | Identity |
| --- | --- | --- |
| *malL* | Oligo-1,6-glucosidase | 74.9 |
| *sacA* | Sucrose-6-phosphate hydrolase | 54.0 |
| *xynA* | Endo-1,4-beta-xylanase A | 57.0 |
| *amyE* | Alpha-amylase | 87.1 |
| *xynB* | Beta-xylosidase | 94.0 |
| *eglS* | Endoglucanase | 92.8 |
| *xynD* | Arabinoxylan arabinofuranohydrolase | 89.8 |
| *xynC* | Glucuronoxylanase XynC | 90.5 |
| *ilvH* | Putative acetolactate synthase small subunit | 98.8 |
| *ilvB* | Acetolactate synthase large subunit | 89.5 |
| *alsD* | Alpha-acetolactate decarboxylase | 82.7 |
| *alsS* | Acetolactate synthase | 83.7 |
| *bdhA_1* | D-beta-hydroxybutyrate dehydrogenase | 70.2 |
| *bdhA_2* | D-beta-hydroxybutyrate dehydrogenase | 78.8 |

**Supplementary Figures**


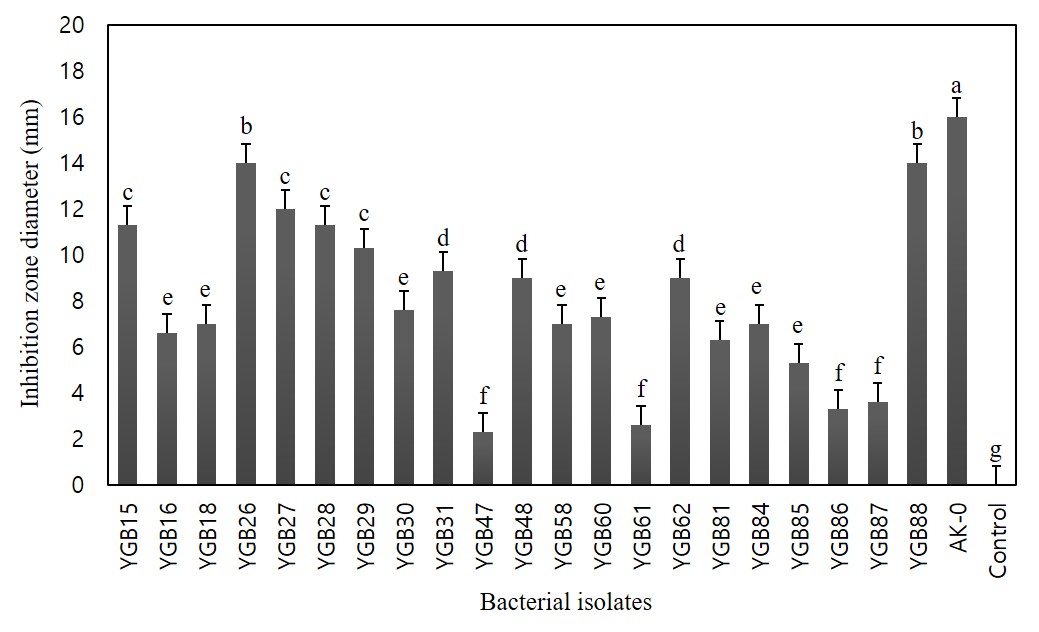


**Figure S1.** ***In vitro* screening of antagonistic activity.** The ginseng rhizospheric soil bacteria were tested for antagonistic activity against *Colletotrichum gloeosporioides,* a fungal pathogen causing anthracnose disease on apple, using a disc diffusion assay. A sterile paper disk (8 mm diameter) with bacterial suspensions (10^8^ cfu/mL) was placed onto the potato dextrose agar (PDA) plate 30 mm away from the pathogenic fungi. The inhibition zone was measured five days after incubating at 25 °C. The experiment was performed two times with five replicates (plates) per treatment with similar results. Bars with the same letters do not differ significantly between each other according to the least significant difference (LSD; *P* < 0.05).


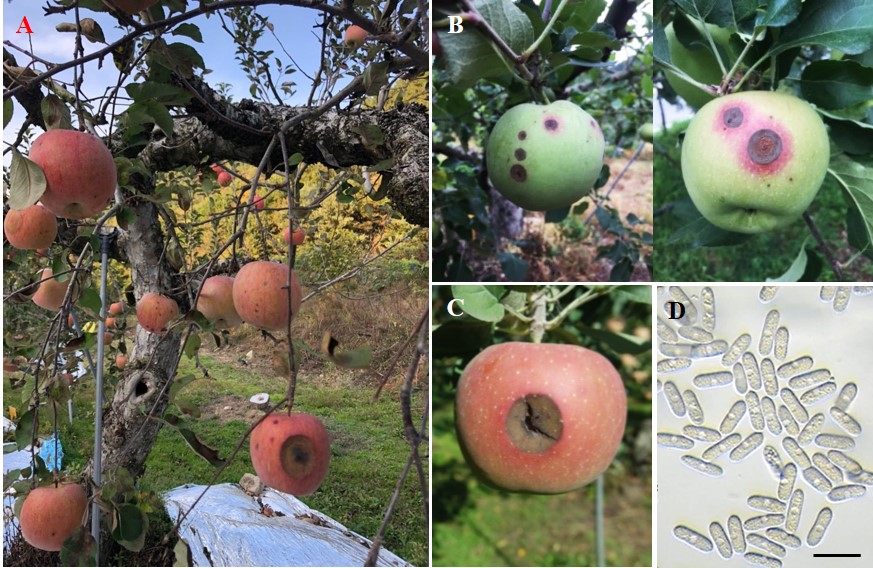


**Figure S2. Disease occurrence of bitter rot caused by *Colletotrichum gloeosporioides* in apple**. (**A**) Apple tree bearing fruits with anthracnose disease symptoms. (**B**) Bitter rot symptoms appear as small circular spots on immature apple and increases in size gradually. (**C**) The spots become brown in color as the fruit matures. (**D**) Microscopic observation of conidia of the pathogen. Bar = 20 µm.


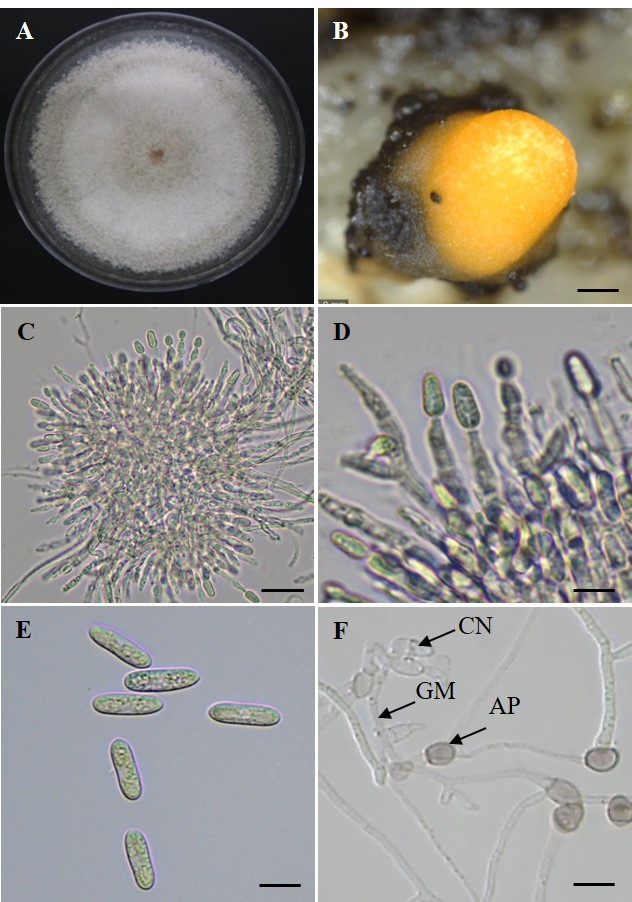


**Figure S3. Isolation and identification of *Colletotrichum gloeosporioides* APEC18-004 causing bitter rot in apple.** (**A**) Morphological characteristics of fungal mycelia of *C. gloeosporioides* APEC18-004 on potato dextrose agar (PDA) plate. (**B**) Conidiomata with conidia (bar = 20 µm). (**C–D**) Conidiophore stalk. (**E**) Separation of conidia from conidiophores. (**F**) Formation of appressorium-like structure by germ tube from conidia. The image was photographed 10 days after incubation at 25 °C on PDA plates. Scale bar = 10 µm. AP: appressorium, GM: germ tube, CN: conidia.


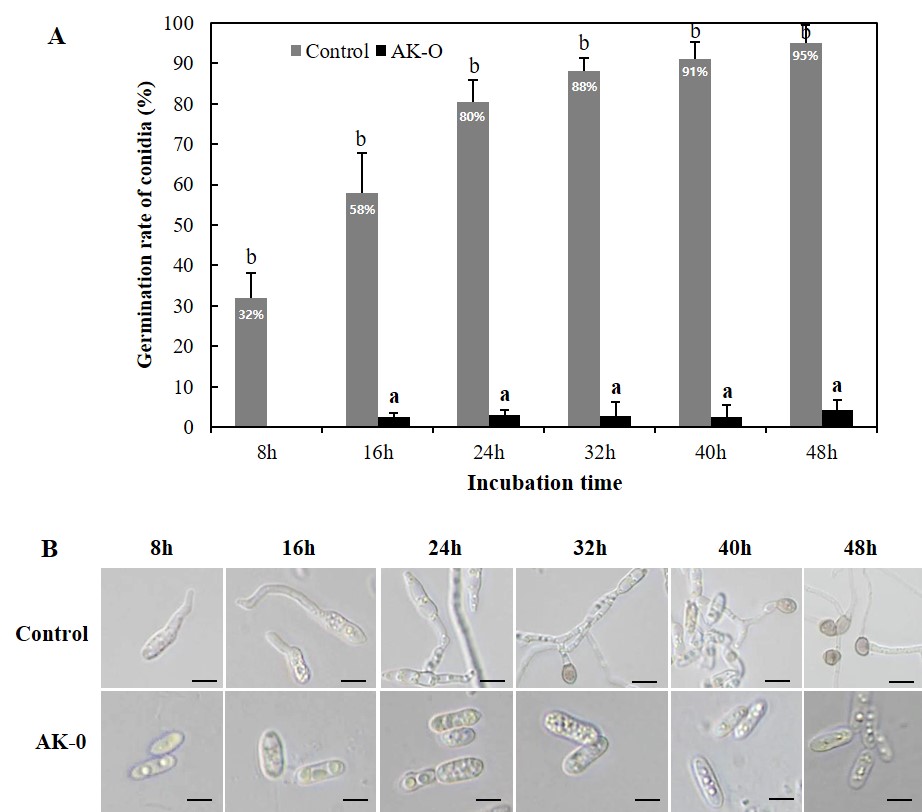


**Figure S4. Effect of AK-0 cell suspensions on conidial germination rate and microscopic observation. (A)** The percentage of conidial germination rate was reduced in the AK-0 treatment compared to that in the non-treated control. **(B)** Microscopic observations of the fungal spore germination of *Colletotrichum gloeosporioides* APEC18-004 after AK-0 treatment during the incubation period from 8 to 48 h. Conidia were germinated at 16 h in the non-treated control. No germination was observed in the AK-0-treated conidia. Germination counting was performed using a hemocytometer. The experiment was repeated three times with three replicates per treatment producing similar results. Bar = 10 µm. The representative image is presented here. Bars with same letters do not differ statistically between the treated sample and the non-treated control, according to the least significant difference (LSD) at *P* < 0.05.


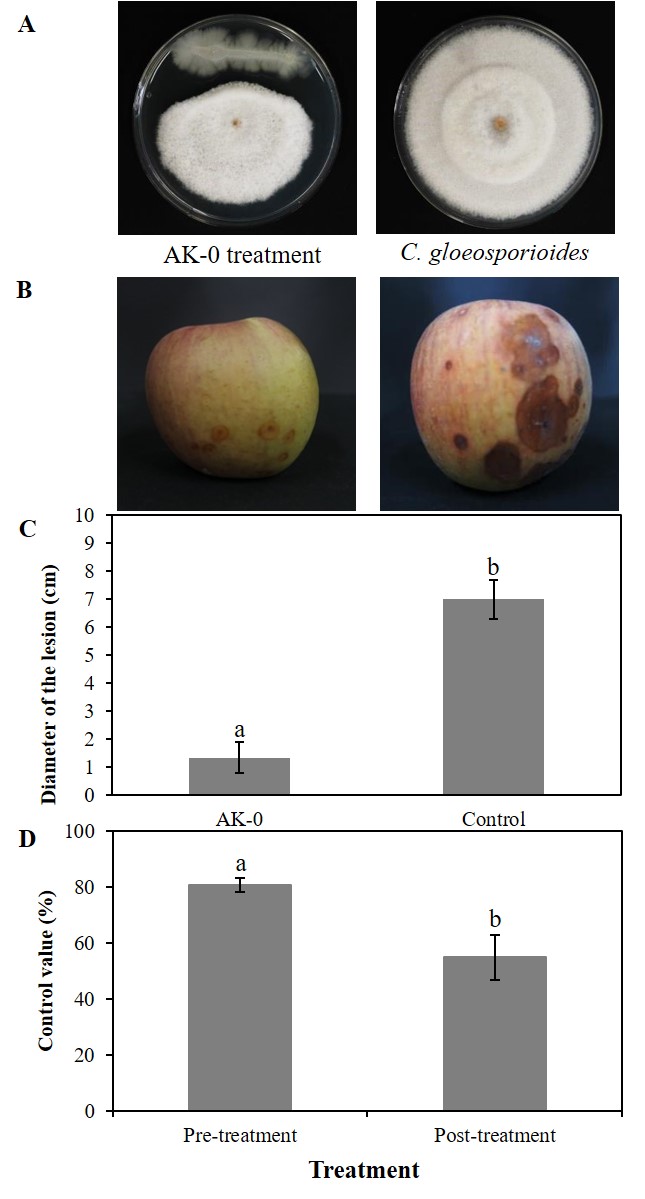


**Figure S5. *In vitro* antagonism, and *in planta* assay on disease suppression of bitter rot on apple caused by *C. gloeosporioides***. (**A**) An *in vitro* antagonistic activity of *B. velezensis* AK-0 against apple bitter rot caused by *C. gloeosporioides* using a confrontation plate assay by streaking the mycelial inoculum onto the center of the potato dextrose agar (PDA) plates. The growth inhibition of mycelia was recorded 7 days after incubation at 25 ℃ and compared to that in the non-treated control. The experiment was performed two times with three replicates. (**B**) The disease severity was reduced in AK-0-treated apples compared to that in the non-treated control. A single image from eight replicates is presented here. (**C**) Diseased lesions were measured in terms of diameter (cm). The diameter of diseased lesion was decreased in AK-0-treated apples compared to that in the non-treated control. (**D**) AK-0 cell suspensions (10^8^ cfu/mL) were applied in apples before (pre-treatment) and after (post-treatment) challenge inoculation with spore suspensions of *C. gloeosporioides*. Disease control values were measured 5 days after inoculation with spore suspensions of *C. gloeosporioides.* Bars with the same letters do not differ each other statistically, according to the least significant difference (LSD) at *P* < 0.05. Each treatment contains 8 replicates (fruits) and the experiment was performed two times.


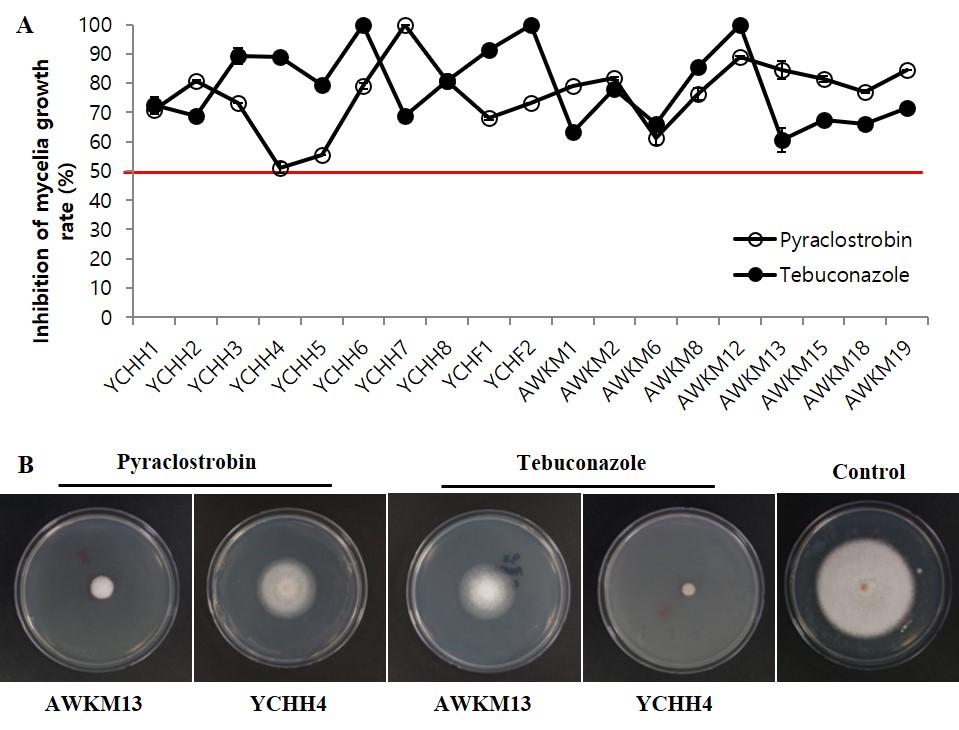


**Figure S6. Screening of fungicide resistant pathogenic strains of *Colletotrichum gloeosporioides.*** (**A**) Selection of fungicidal resistant strains of *C. gloeosporioides* fungal pathogens against chemical fungicides of pyraclostrobin with 20% wettable dispersible granules (WG) at 335 ppm and tebuconazole with 25% wettable powder (WP) at 1000 ppm were used on potato dextrose agar (PDA) plates. (**B**) Two fungal strains (YCHH4 and AWKM13) selected as resistant to fungicides pyraclostrobin and tebuconazole, respectively, on PDA plates in comparison to the no-antifungal control. Each treatment contained three replicates (plates) and the experiment was performed two times. Graph was drawn from the mean values of two independent experiments.


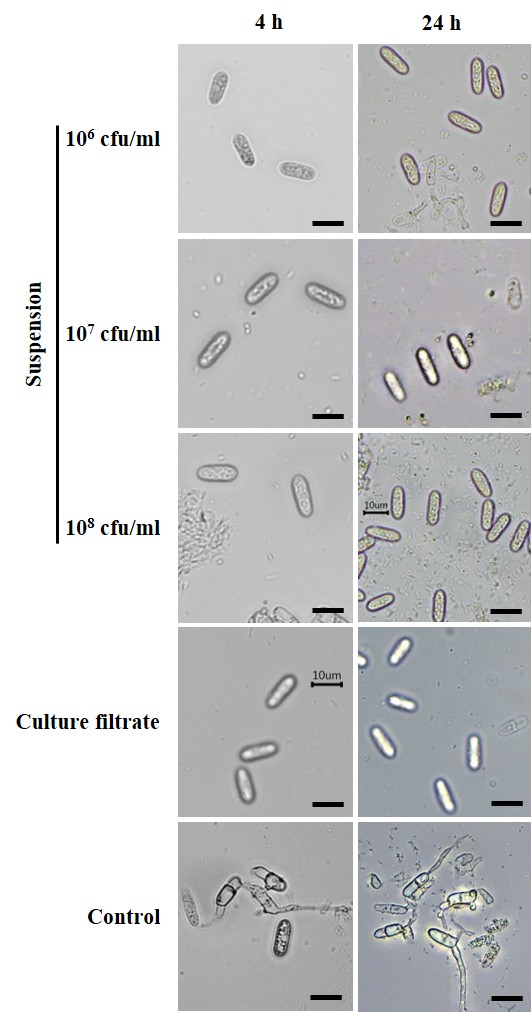


**Figure S7.** Effect of AK-0 cell suspensions at various concentrations (10^6^ or 10^7^ or 10^8^ cfu/ml) and culture filtrate (CF) on the germination of conidia of *Colletotrichum gloeosporioides* YCHH4, a fungicide-resistance fungal pathogenic isolate after 0–24 h incubation at 25 °C in comparison to the non-treated control. Bar = 10 µm.


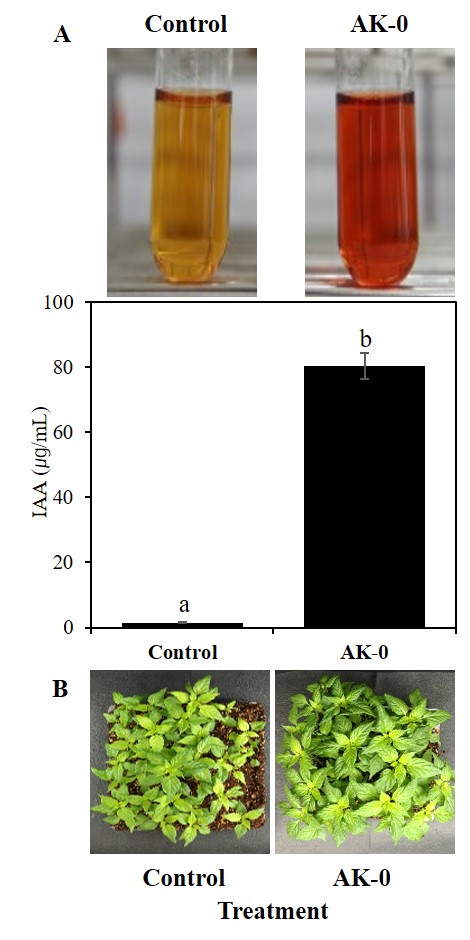


**Figure S8. Effect of AK-0 on plant growth promotion.** (A) The plant growth regulator indole-3-acetic acid (IAA) was detected in AK-0 (observed as pink color development by salkowski reagent), while there was no pink color development in the non-treated control because of no addition of AK-0 cells. (B) hence IAA was not detected (**A**), and the soil drench of two-week-old red-pepper seedlings with AK-0 cell suspensions (1 × 10^6^ cfu/mL) showed an enhanced growth in terms of plant height (**B**). The experiment was performed two times, with 36 replicates (seedlings) in each treatment. Bars with the same letters do not differ statistically between the treated sample and the non-treated control, according to the least significant difference (LSD) at *P* < 0.05.


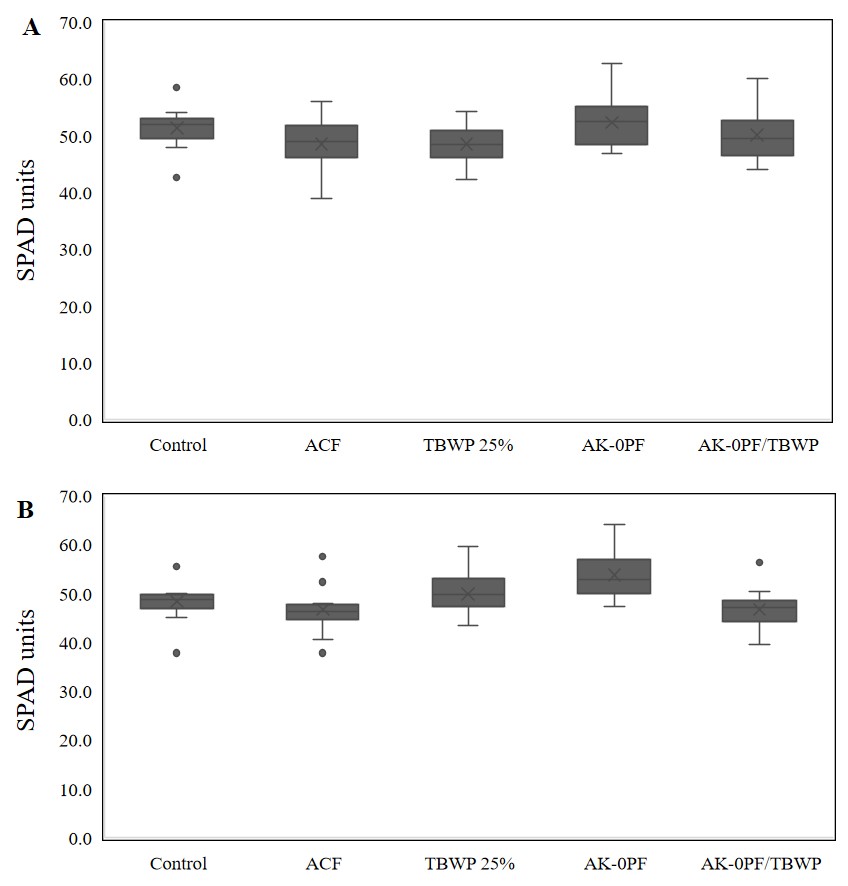


**Figure S9.** Effects of AK-0PF and chemical fungicides on the chlorophyll contents of apple leaves using a SPAD-502 meter in Yecheon (**A**) and Mungyeong (**B**). Readings were taken from the leaves of one-year-old branches and from 20 leaves per tree, with three replications every two weeks.


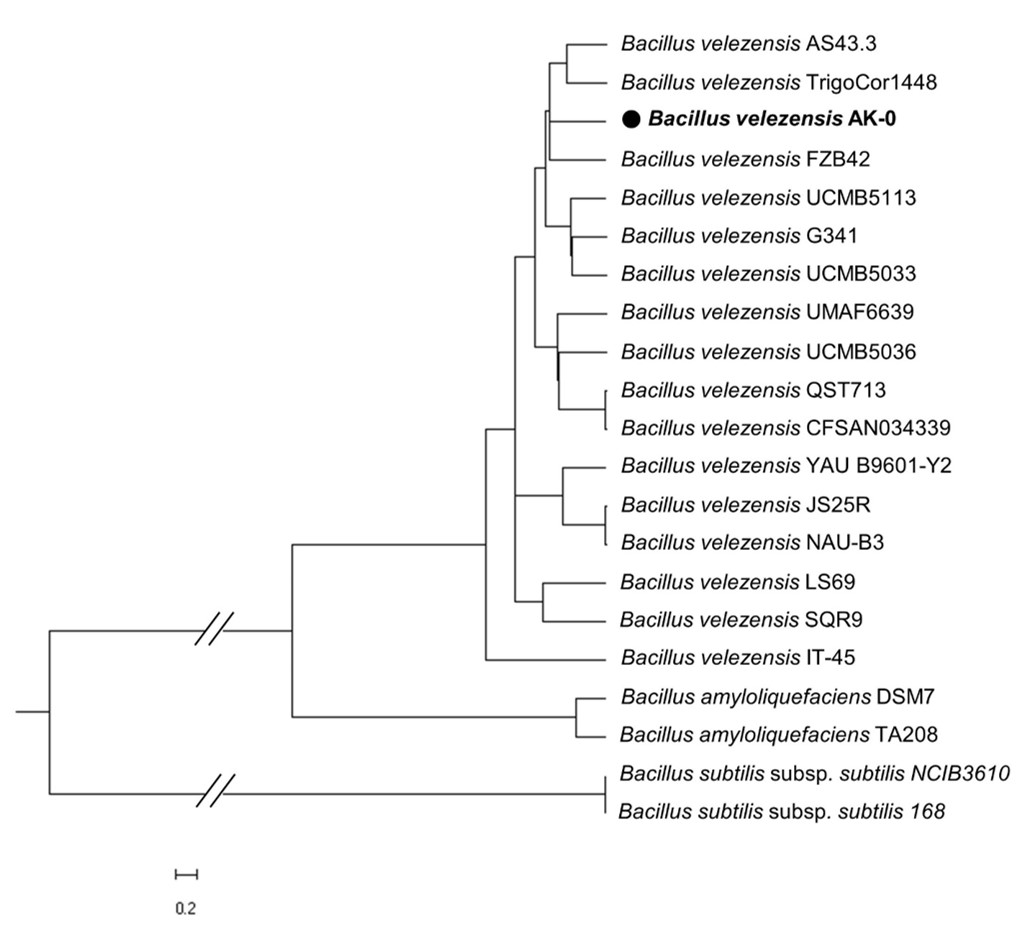


**Figure S10**. Neighbor-joining phylogenetic tree constructed from the core genomes of 21 *B. velezensis* and *B. subtilis* strains. Unweighted Pair Group Method with Arithmetic Mean (UPGMA) dendrogram of the analyzed *Bacillus* spp. based on the orthologous Average Nucleotide Identity (Ortho ANI) value (Lee *et al*., 2016)^27^. The position of *Bacillus velezensis* AK-0 in the tree is marked by a black dot in bold.


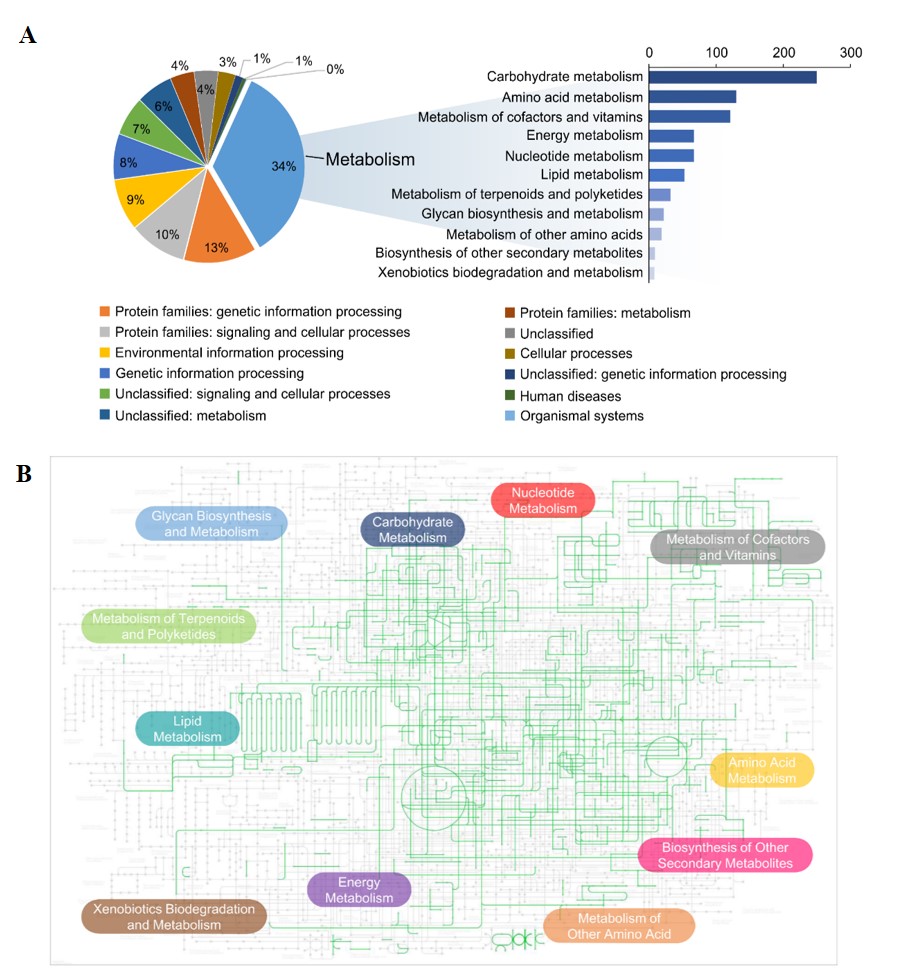


**Figure 11.** Metabolic connection analysis of the changed metabolic modelling in AK-0. (A) Metabolic network of the differential metabolites and altered metabolic pathways in the KEGG general metabolic pathway map. Green lines represent the increased metabolites in AK-0. (B) The KEGG metabolic map overlaid with gene expression data (green lines: gene expressions). The structure of the metabolic network. In this map, each dot represents an intermediate; each line represents an enzyme that acts on an intermediate. The original general metabolic pathway map is available at https://pathways.embl.de/ipath3.cgi. KEGG, Kyoto Encyclopedia of Genes and Genomes; SMPDB, Small Molecule Pathway Database; FZB42, *Bacillus velezensis* AK-0*.*
